# Structural and Thermodynamic Properties of RNA Molecules Using a Knowledge-Based Model

**DOI:** 10.1021/acs.jctc.6c00491

**Published:** 2026-07-13

**Authors:** Mario Villada-Balbuena, Mauricio D. Carbajal-Tinoco

**Affiliations:** † Departamento de Física, Centro de Investigación y de Estudios Avanzados del IPN, Av. Instituto Politécnico Nacional No. 2508, Col. San Pedro Zacatenco, CP 07360 Ciudad de México, Mexico; ‡ Escuela de Ingeniería y Ciencias, 27746 Tecnologico de Monterrey, Av. Eugenio Garza Sada 2501, CP 64849 Monterrey, Nuevo León, Mexico

## Abstract

We present a coarse-grained model that describes the unfolding process and thermodynamics of ribonucleic acid (RNA) molecules. We obtained and analyzed a set of 1944 three-dimensional RNA structures of various molecular weights and under diverse conditions from the Protein Data Bank. We reduced the description of these molecules from an all-atom representation to a single interacting point per nucleotide, located at its center of mass. From this information, we calculated characteristic properties of the RNA chains, such as the bond distribution function and the contour length, which allowed us to estimate the most probable distance between two nucleotides linked by a phosphodiester bond as *a* = 5.5 ± 0.4 Å. We also calculated the radius of gyration of these chains, through which we obtained an estimate of the Flory exponent, ν = 0.33 ± 0.01, and a fractal dimension, *d*_F_ = 3.03 ± 0.09. Furthermore, we determined the persistence length to be *l*_p_ = 9.5 ± 4.1 Å. On the other hand, the different molecular configurations were used to improve the statistics of the pair distribution functions for various degrees of freedom. These were employed to obtain effective interaction potentials in a previous model [Villada-Balbuena, M.; Carbajal-Tinoco, M. D. *J. Chem. Phys.* **2024**, 161, 165104.], which underwent a series of improvements, reducing the number of fitting parameters and enhancing the description of the radial-angular interaction. The fitting parameters of these potentials were optimized through Brownian dynamics (BD) simulations using the iterative Boltzmann inversion algorithm. The optimized potentials were used in steered BD simulations to model the mechanical unfolding at a constant velocity of a series of hairpins and pseudoknots. The results of these simulations are contrasted with experimental data, achieving excellent agreement. During the unfolding process, we monitored the configurational temperature (CT) of the model’s different degrees of freedom as well as the total CT. We used Jarzynski’s equality to calculate the Helmholtz free energy change, Δ*A*. Through Δ*A* and the integral of the force–extension curve, we obtained the Gibbs free energy change Δ*G*, which was successfully compared with the experimental results of RNA molecules unfolding using optical tweezers. Finally, based on the internal energy change values from the simulations, we estimated the entropy change Δ*S*. These values were compared with entropy changes from theoretical models. Finally, we utilized our model to calculate the changes in the aforementioned thermodynamic functions for molecules associated with viral protein expression.

## Graphical abstract

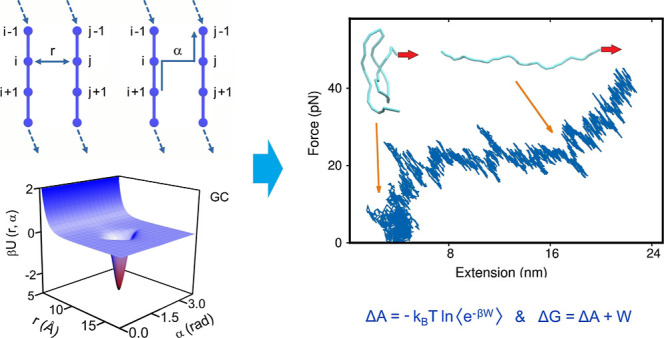

## 1 Introduction

Ribonucleic acid (RNA) molecules have an essential role in a wide array of cellular biological processes.^1^ In addition to the roles they play in well-known functionalities, new processes that these molecules can perform continue to be discovered, such as self-replication and the synthesis of their complementary strands.^2^ On the other hand, long noncoding RNAs can be synthesized outside cells, engineered into stable therapeutics, and used to tamp down acute inflammation in mice and human immune cells.^3^ Although the relationship between three-dimensional (3D) structure and RNA function is widely accepted, it remains an active area of research.^4^ Consequently, determining the structure of RNA molecules is of paramount importance. In this regard, cryogenic electron microscopy (Cryo-EM) has emerged as one of the most rapidly advancing techniques in recent years.

Cryo-EM has revolutionized the acquisition of atomic-scale 3D structures in near-native states without the need for molecular crystallization. This technique has been employed to characterize both proteins and nucleic acids.^5^ Unlike other methods, Cryo-EM enables the structural determination of very large molecular assemblies, as evidenced by the increasing number of RNA structures deposited in databases such as the Protein Data Bank (PDB)^6^ and the Nucleic Acid Knowledgebase.^7^

From these 3D structures, characteristic lengths of RNA molecules, such as contour length, radius of gyration, and persistence length,^8^ can be derived.^9^ Through theoretical models, these microscopic features can be linked to macroscopic properties. In this context, a groundbreaking class of experiments involves measuring microscopic quantities and relating them to macroscopic properties via mechanical unfolding using optical tweezers.^10,12^ When these experiments are conducted near equilibrium,^13^ the change in the Gibbs free energy can be calculated from the force applied to the molecule and the resulting end-to-end displacement. For nonequilibrium conditions, theoretical tools such as the Jarzynski equality can be applied to estimate changes in the same thermodynamic function.^14^

In addition to optical tweezer experiments, numerical models and tools have been developed for computational simulations of mechanical unfolding. Two such approaches are the Three Interaction Sites (TIS)^15^ and the Self-Organized Polymer (SOP)^16^ models. In these coarse-grained models, nucleotides (nts) are represented either by three interacting sites (phosphate, ribose, and nitrogenous base) or by a single interacting site per nucleotide. These models are computationally less demanding than numerical simulations such as density functional theory or all-atom molecular dynamics (MD), which typically have simulation time scales limited to hundreds of picoseconds or nanoseconds-to-microseconds, respectively.^17^ In contrast, the experimental stretching of an RNA molecule can reach time scales on the order of 1 s.^10^ The TIS and SOP models have been used to simulate the mechanical unfolding of structures ranging from simple 22 nt hairpins with both models (e.g., PDB ID: 1F9L)^15,18^ to complex ribozymes with the SOP model.^19^

Despite their utility, these models have their limitations. In the SOP model, for instance, oscillations in force–extension curves (FECs) are smaller than those observed experimentally, suggesting that the simulated structure may be overstabilized. This behavior stems from the radial symmetry of the attractive potential between nucleotides, which ignores the relative orientations of the interacting sites. Furthermore, the SOP model primarily considers native-state attractive interactions, limiting its ability to simulate regional rearrangements or changes in the plateau slope of FECs, phenomena frequently observed in experimental unfolding.^10,13^

Similar to in singulo mechanical unfolding experiments, microscopic properties can be quantified using MD and Brownian dynamics (BD) simulations. By applying the Jarzynski equality,^14^ macroscopic properties can be derived. While MD simulations can be performed in various ensembles (e.g., canonical, isobaric–isothermal, or a combination of both^20^), allowing for the calculation of both Helmholtz and Gibbs free energy changes, BD simulations utilizing integration algorithms such as Ermak-McCammon^21^ or Iniesta-García de la Torre^22^ are typically restricted to the canonical ensemble. Consequently, BD primarily quantifies Helmholtz free energy. However, since experiments typically measure Gibbs free energy changes, classical thermodynamics allows us to relate the two through the work applied by the optical tweezers,^23^ enabling direct comparison between BD simulations and experimental data. Beyond free energies, entropy is a thermodynamic function of significant theoretical interest due to its fundamental link to the partition function,^24^ which provides a complete thermodynamic description of the system. Nevertheless, quantifying entropy changes in molecular systems like RNA remains a challenging research topic.^25^

We present an updated review of the aforementioned characteristic lengths for RNA molecules in the PDB. We revisit a new parameterization of the coupling angle within a previously reported model,^26^ significantly improving mechanical unfolding simulation results and reducing the number of fit parameters. We demonstrate the mechanical unfolding of several experimentally well-characterized molecules,^10,13^ calculating the Helmholtz free energy change via the Jarzynski equality, the Gibbs free energy change,^23^ and the entropy change. Our results show improved performance over previous models with thermodynamic calculations in very good agreement with experimental values. Furthermore, the entropy changes are of the same order of magnitude as the absolute value of the results of theoretical models^8^ and other methods for calculating configurational entropy.^25^ Finally, we provide predictions for the mechanical unfolding of RNA molecules associated with viral diseases in humans, including the SARS-CoV-2 virus.^27^

## 2 Materials and Methods

### 2.1 Obtaining Experimental Structures

Based on available data from the PDB,^6^ we selected a set of 1944 RNA molecules with lengths ranging from 3 to 4298 nucleotides. Their experimental three-dimensional structures were obtained under various conditions, such as pH = 7 ± 1 and temperatures around *T* = 293 ± 7 K. RNA–protein complexes were not excluded from our analysis; for all structures examined, the protein content is approximately 10% by mass. Furthermore, aside from the pH restriction, no selective cutoff was applied based on ionic concentration or specific ion types, as our objective is to characterize an average structural behavior. The outcome of a specific ion can be incorporated through suitable interaction potentials, as was done to model the effect of the Mg^2+^ ion.^28^ Additionally, the average resolution of these structures is 3.1 ± 1.1 Å, and both nonhomologous and homologous structures were selected; the latter, when appropriately weighted, reduce statistical noise in the correlation functions by providing slightly different configurations.

Figure 1 shows a log–log frequency plot of the number of nucleotides in the RNA molecules studied. A similar analysis has been reported previously;^9^ however, due to advances in characterization techniques such as Cryo-EM, the number of high-molecular-weight RNA structures has increased significantly. It is worth mentioning that the number of nucleotides obtained from the PDB file is often slightly lower than the size reported on the platform itself because, in some cases, not all nucleotides can be resolved by the experimental methods used. Below, we present an analysis of certain geometric properties that are useful for describing these molecules.

**Figure 1:**
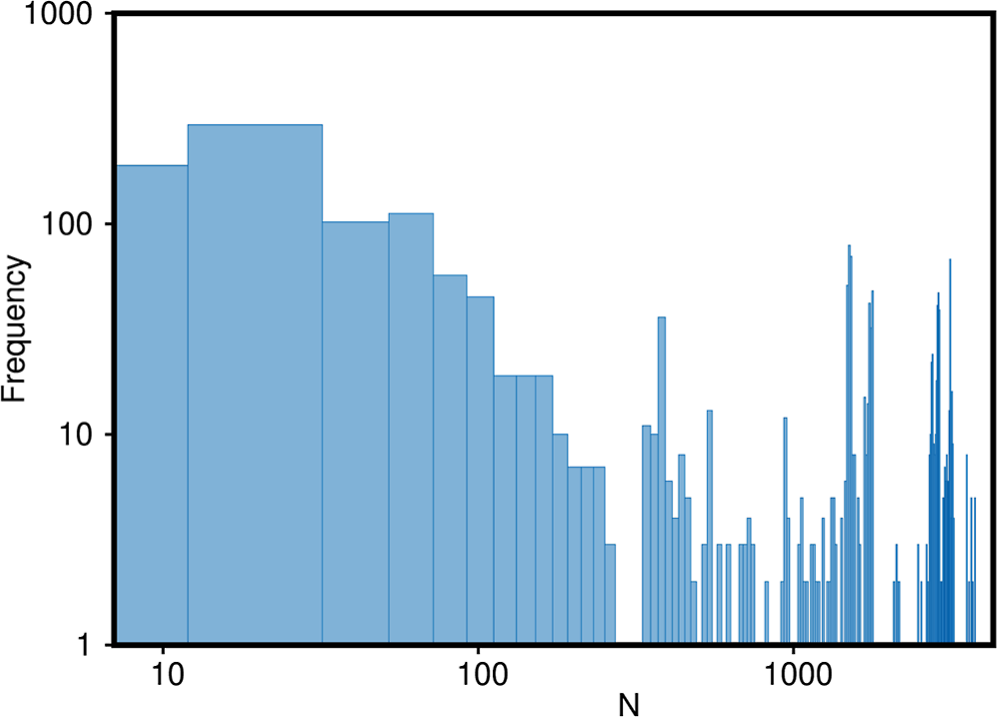
Log–log plot of the frequency distribution (Frequency) of the number of monomers *N* in the analyzed RNA molecules.

### 2.2 Characteristic Lengths of RNA Chains

Generally, the structural properties of polymers are presented in terms of the bond distance between their constituent monomers.^8^ In the description used here, nucleotides (which consist of a phosphate group, a sugar, and a nitrogenous base, either adenine (A), cytosine (C), guanine (G), or uracil (U)) are represented by a single interacting point located at their center of mass. One of the characteristic lengths that depends on the distance between monomers is the contour length *L*_c_, defined as

1 $$L_{c}=\sum_{i=1}^{N-1}\left|r_{i+1}-r_{i}\right|$$

where **r**_*i*_ is the position of the *i*-th monomer. The contour length was calculated for the group of selected RNA molecules. From these results, a linear fit of *L*_c_ as a function of the number of nucleotides in the chains *N* was performed, in the form $L_{c}=\mathrm{a̅}\left(N-1\right)$ , where a̅ is the average distance between monomers. From this fit, a value of a̅ = 5.5 ± 0.002 Å was obtained, with a coefficient of determination *R* ^2^ = 0.9995. The results for *L*_c_ and its linear fit can be seen in Figure 2a. The structures that deviate from the fit line are those that are more incomplete in the PDB. Another way to estimate the average distance between neighboring nucleotides is through the bond distance distribution function.^28^ Using the set of structures, we calculated the pair distribution function *g*(*a*). Since *a* is a local degree of freedom, its pair distribution function is invariant with respect to the size of the RNA chains used to calculate it; that is, the result is the same whether we consider all analyzed molecules or only high-molecular-weight ones. Figure 2b shows the result of *g*(*a*). The line represents the average, while the shaded region indicates the standard deviation. The peak of *g*(*a*) is located at *a* = 5.0 ± 0.1 Å. If we consider not only the maximum value of *g*(*a*) but also the distribution of values, we can calculate the weighted mean distance, obtaining *a* = 5.4 ± 0.7 Å. Finally, we can consider the reported experimental value for the interphosphate distance in the chain backbone, 5.9 Å.^10^ With these data, we can calculate an average value, obtaining *a* = 5.5 ± 0.4 Å, which is consistent with the fitted value from Figure 2a. In addition to the distance between monomers in a polymer chain, another measurement that provides information on the size of RNA chains is the radius of gyration *R*_g_, defined as

2 $$R_{g}^{2}=\frac{\sum_{i=1}^{N}m_{i}x_{i}^{2}}{\sum_{i=1}^{N}m_{i}}$$

where *m*_*i*_ is the mass of the *i*-th monomer and **x**_*i*_ is the position of that monomer relative to the center of mass, **x**_*i*_ = **r**_*i*_ – **R**_CM_ with $R_{CM}=\sum_{i=1}^{N}m_{i}r_{i}/\sum_{i=1}^{N}m_{i}$ . The radius of gyration was calculated for the set of structures obtained from the PDB. From these data, a fit of the form *R*_g_ = *aN* ^ν^ was performed,^8^ where *a* is the distance between monomers and *N* is the degree of polymerization. Using the previous characteristic distance values, we obtain an average Flory exponent of ν = 0.33 ± 0.01. This result is similar to those previously reported.^9,29^ From the value of ν, the fractal dimension was calculated *d*_F_ = 1/ν,^30^ resulting in *d*_F_ = 3.03 ± 0.09.

**Figure 2:**
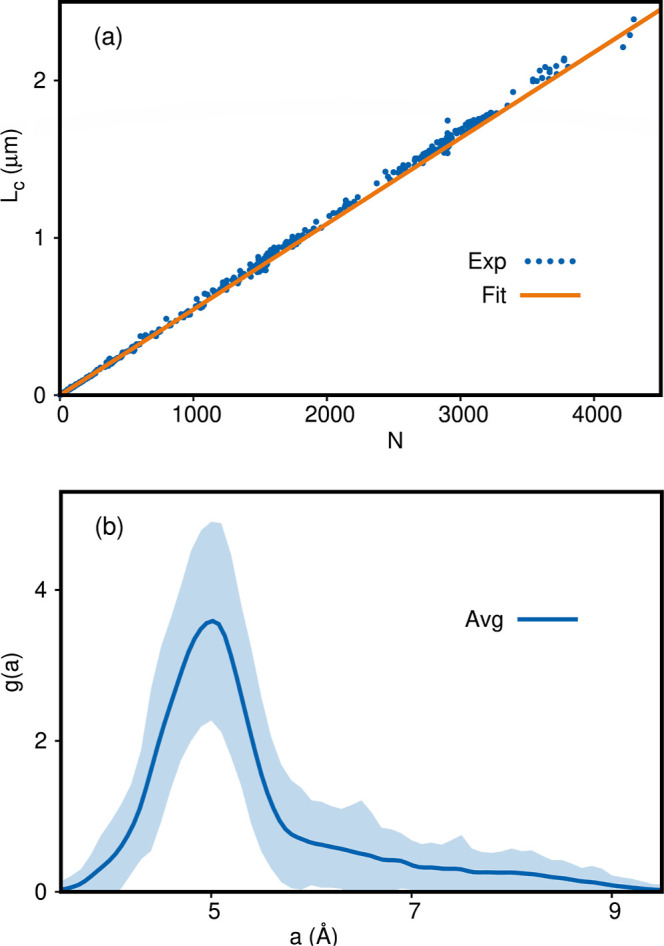
(a) Contour length *L*_c_ of RNA molecules as a function of the number of nucleotides *N*; the line is the least-squares fit to the function $L_{c}=\mathrm{a̅}\left(N-1\right)$ , where a̅ is the mean distance between monomers with a value of a̅ = 5.5 ± 0.002 Å. (b) Bond distribution function, *g*(*a*), obtained from the analysis of 1944 RNA molecules from the PDB. Here, *a* is the separation between monomers. The line represents the average, while the shaded region is the standard deviation of the experimental data.

Figure 3a shows the radii of gyration of the analyzed RNA chains and the performed fit. There is a subset of seven molecules with radii of gyration that deviate from the trend; these molecules are characterized by having very large incomplete regions in the PDB file. It is worth noting that as the number of nucleotides increases, the radius of gyration tends to decrease relative to the average line; this may be because larger molecules have a greater number of interactions between nucleotides, causing the volume to grow more slowly than the mass, which is directly related to the number of monomers in the molecule. Another characteristic length is the persistence length, *l*_p_, which measures the rigidity (or flexibility) of the molecule. This can be estimated through a fit to the expression^31^

3 $$\left\langle {â}_{i}\cdot {â}_{j}\right\rangle =\exp\left(-L_{c}/l_{p}\right)$$

where |**a**_*i*_| is the distance between particles *i* and *i* – 1, **a**_*i*_ = **r**_*i*_ – **r**_*i*–1_, and ${â}_{i}$ is the unit vector ${â}_{i}=a_{i}/\left|a_{i}\right|$ . In Figure 3b, we observe that the data dispersion is very large for small molecules, but as the number of monomers increases, *l*_p_ appears not to vary as a function of *N*, as suggested in other studies.^9^ The average persistence length for all analyzed chains is *l*_p_ = *a*(1.74 ± 0.76) = 9.5 ± 4.1 Å, for *N* ≥ 3. This high uncertainty stems from the significant numerical dispersion of *l*_p_ for very short RNA chains, as previously mentioned. Nevertheless, the standard deviation decreases rapidly as N increases. Thus, *l*_p_ = 9.5 ± 1.0 Å, for *N* ≥ 30, and *l*_p_ = 9.5 ± 0.5 Å, for *N* ≥ 60. Specifically, *N* ≥ 31 for the cases analyzed in this work. The main value of *l*_p_ is similar to what has been previously reported.^32^

**Figure 3:**
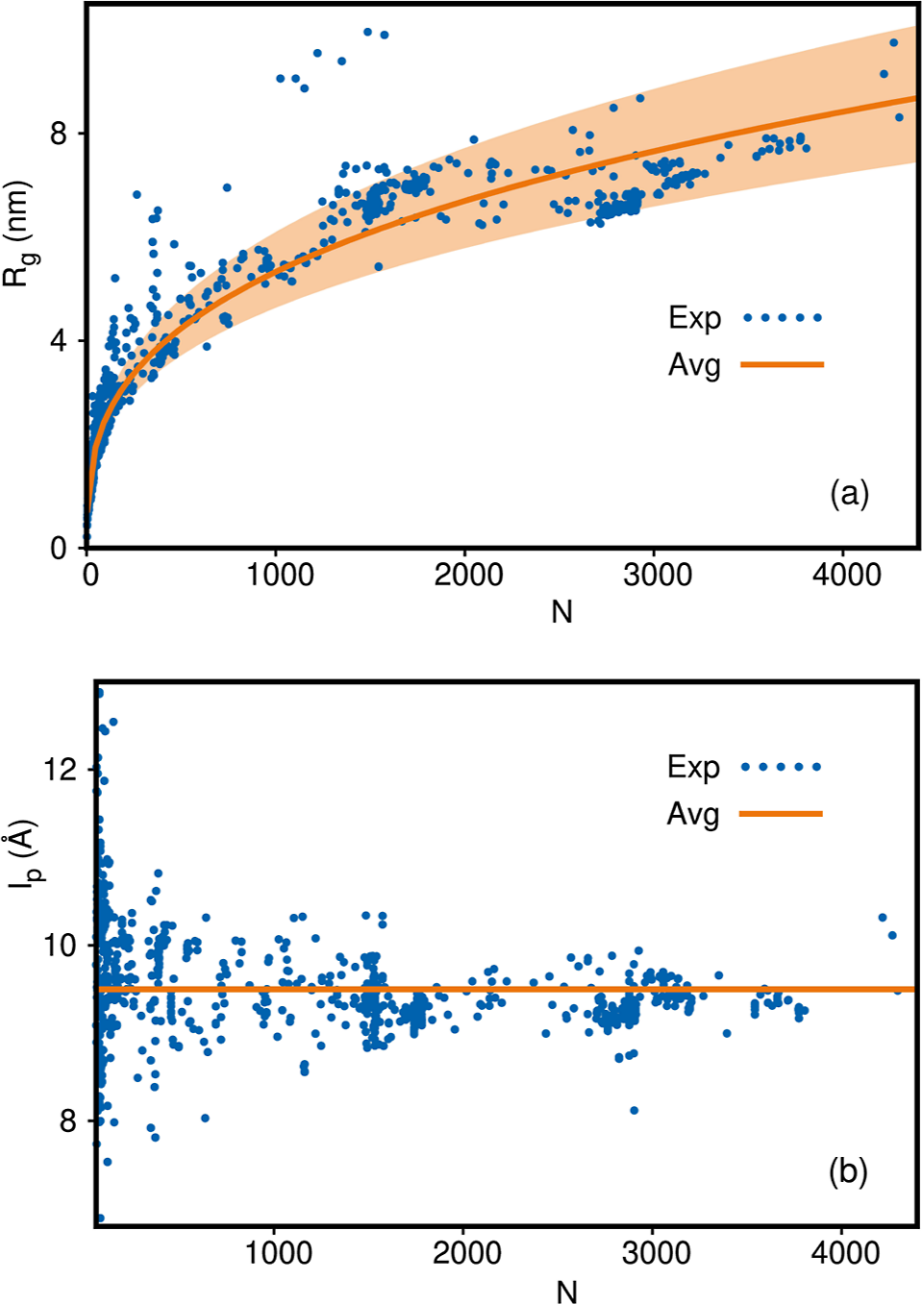
(a) Radius of gyration *R*_g_ of RNA molecules as a function of the number of nucleotides *N*. The solid line represents the fit of the form *R*_g_ = *aN* ^ν^ with *a* = 5.5 Å, ν = 0.33, while the light orange region shows the variations of the fitting parameters. (b) Persistence length *l*_p_ as a function of the number of nucleotides *N*. The solid line represents the average of the persistence length.

### 2.3 Simpler Effective RNA Interaction Model

From the set of structures obtained from the PDB, we calculated the distribution functions of the local degrees of freedom: bond (*a*), bending (θ), and torsion (ϕ). In the case of nonlocal interactions, only structures with a nucleotide count greater than 1000 nt were considered to calculate the distribution functions depending on the separation distance between nucleotides (*r*) and a coupling angle (α), resulting in a total subset of 818 large structures. The definition of the degrees of freedom has remained the same as in previous works,^26,28^ except for the coupling angle, which has changed to an expression that is simpler to apply and offers computational advantages, given that it now depends on only 4 particles instead of 6.

At this stage, we introduce the concept of an effective interaction potential, the purpose of which is to reproduce its corresponding pair distribution function. Consequently, such a potential incorporates the integrated effect of various short- and long-range interactions, including the influence of other nucleotides, solvent molecules, ions in solution, or even surrounding proteins. Prior to the determination of effective interaction potentials, Figures S1, S2, and S3 of the Supporting Information Section illustrate three examples of the convergence of pair distribution functions for all the different combinations of nucleotides toward the average curve for each of the corresponding degrees of freedom.

From the distribution functions, we calculated a first approximation of the effective potential using the potential of mean force (PMF) approximation^33^

4 $$\beta U_{\mu \nu }\left(\xi \right)=-\ln\left[g_{\mu \nu }\left(\xi \right)\right]$$

where β^–1^ = *k*_B_ *T* is the thermal energy; *k*_B_ is the Boltzmann constant; *T* is the absolute temperature; μ and ν represent A, C, G, or U; and ξ represents the degrees of freedom *a*, θ, ϕ, or *r*. Therefore, *g*_μν_(ξ) is the pair distribution function of species μ and ν, for the degree of freedom ξ. For the most part, the functions used to fit the effective interaction potentials have remained invariant.^26^ However, two changes have been made. On the one hand, the fitting function for the bond potential has been modified as follows. Since the FENE (Finitely Extensible Nonlinear Elastic) potential can lead to numerical divergences due to the presence of a logarithmic function, it was replaced by a simpler functional form such as the harmonic potential, which also allows us to correctly model variations around the equilibrium position, while leaving the Gaussian well unaltered

5 $$U\left(a\right)=\frac{1}{2}K_{0}{\left(a-a_{0}\right)}^{2}-A_{b}\;\exp\left[-\frac{1}{2}{\left(\frac{a-a_{1}}{\sigma_{b}}\right)}^{2}\right]$$

where *a* = |**a**_*i*+1_| = |**r**_*i*+1_ – **r**_*i*_|, with **r**_*i*_ being the position of the *i*-th nucleotide, *K*_0_ quantifies the bond stiffness, *a*_0_ is the equilibrium distance of the harmonic potential, and *A*_b_, *a*_1_, and σ_b_ are the depth, equilibrium distance, and half-width of the Gaussian well, respectively.

The second modification made to the model is the definition of the coupling angle, α. Now, α is defined as follows: the position of nucleotide *i* is given by **r**_*i*_, and the vector joining two adjacent nucleotides *i* and *i* + 1 is **a**_*i*+1_ = **r**_*i*+1_ – **r**_*i*_. The coupling angle between two nucleotides *i* and *j*, with *j* ≥ *i* + 4, is calculated as

6 $$\alpha =\pi +\arctan2\left[\left(\left(a_{i+1}\times r_{ij}\right)\times \left(r_{ij}\times a_{j}\right)\right)\cdot r_{ij}/\left|r_{ij}\right|,\left(a_{i+1}\times r_{ij}\right)\cdot \left(r_{ij}\times a_{j}\right)\right]$$

where **r**_*ij*_ = **r**_*j*_ – **r**_*i*_ and α∈[0,2π). In this way, the coupling angle goes from depending on the position of 6 particles to only 4. This definition represents an angle similar to the torsion angle ϕ, but between nucleotides *i* + 1, *i*, *j*, and *j* – 1 (see Figure 4b). It is worth mentioning that all possible combinations between nucleotides *i* – 1, *i*, *i* + 1, *j* – 1, *j*, and *j* + 1 were explored, and we kept the one that generated the radial-angular distribution functions with the highest maximum. With this definition of α, the function used to model the experimental results is simplified to

7 $$U_{\mu \nu }\left(r,\alpha \right)={\left(\frac{A_{\mu \nu }}{r}\right)}^{8}-B_{\mu \nu }\left[\frac{\sigma_{\mu \nu }^{2}}{{\left(r-d_{\mu \nu }\right)}^{2}+\sigma_{\mu \nu }^{2}}\right]\exp\left[-\frac{1}{2}{\left(\frac{\alpha -e_{\mu \nu }}{s_{\mu \nu }}\right)}^{2}\right]$$

where *r* = |**r**_*j*_ – **r**_*i*_|, *A*_μν_ modulates the stiffness of the potential barrier, *B*_μν_ controls the depth of the Gaussian well depending on the coupling angle, *e*_μν_ is the half-width of the Gaussian potential well of the coupling angle, *d*_μν_ is the equilibrium distance, and σ_μν_ is the half-width of the distance-dependent potential well.

**Figure 4:**
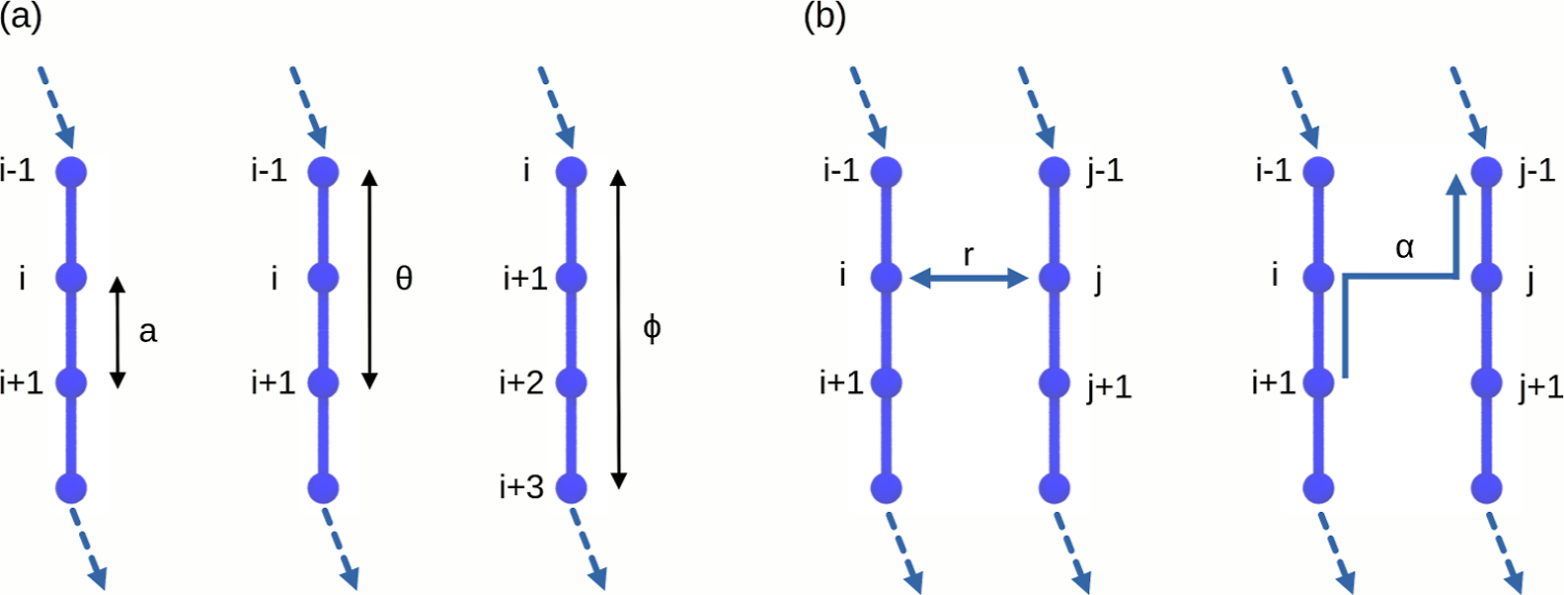
Representation of the model’s degrees of freedom. Each sphere represents the center of mass of a nucleotide. (a) Local degrees of freedom: bond (*a*), bending (θ), and torsion (ϕ). (b) Nonlocal degrees of freedom: separation distance (*r*) and coupling angle (α).

Thus, the total potential energy function between nucleotides takes the form

8 $$U_{\mu \nu }=U\left(a\right)+U\left(\theta \right)+U\left(\phi \right)+U_{\mu \nu }\left(r,\alpha \right)$$

As can be noted from the previous equation, the values of the fitting parameters for the local potentials are invariant with respect to the type of nucleotides, whereas in the radial-angular potential, the parameters depend on the species of the combinations being considered. A schematic representation of the model’s degrees of freedom is shown in Figure 4. The expressions for the interaction potentials can be found in the Supporting Information Section.

Although the new definition of the coupling angle is simpler, the fits to the width of the potential well in its angular part are considerably better than those in previous versions. Additionally, in the mechanical unfolding simulations, the frequency of events where RNA chains transition from the folded to the unfolded state without exhibiting continuous unfolding increased, going from a success rate of less than 30% to more than 80%. Both modifications have allowed for a reduction from 83 to 72 fitting parameters (20 additional parameters are required to describe the interaction of nucleotides with magnesium ions).

For the radial potentials, the distribution function is corrected due to finite-size effects. We start with a test sphere with radius *r*_max_ and volume *V* = 4π*r*_max_^3^/3 containing nucleotides of species μ and ν. For a distance *r*, the distribution function *g*_μν_(*r*) is given by^34^

9 $$g_{\mu \nu }\left(r\right)=\frac{NV}{N_{\mu }N_{\nu }}\left(\frac{h^{\prime }\left(r\right)}{N4\pi r^{2}dr-N^{\prime }V_{c}\left(r\right)}\right)$$

where *N*_μ_ and *N*_ν_ are the number of particles of species μ and ν within volume *V*, *N* = *N*_μ_ + *N*_ν_, and *h*′(*r*) is the total number of nucleotides of the same species between two concentric spheres of radii *r* and *r* + *dr* around a central nucleotide. If the central nucleotide is located within the sphere of radius *r*_max_ – *r*, then *V*_c_ = 0 (denoted as region I). Otherwise, for a nucleotide outside region I but still within the test sphere (region II)

10 $$V_{c}=\pi \left[\left(r_{\max}^{2}-r^{2}\right)\ln\left(1-\frac{r}{r_{\max}}\right)+\frac{3}{2}r^{2}+rr_{\max}\right]dr$$

where *N*′ in eq 9 is the number of nucleotides within region II. The geometry of the system can be consulted elsewhere.^35^ The inclusion of this degree of freedom adds an angular dependence to the radial distribution function as follows:

11 $$g_{\mu \nu }\left(r,\alpha \right)=\frac{g_{\mu \nu }\left(r\right)}{\rho_{\alpha }d\alpha }$$

where *g*_μν_(*r*) is given by eq 9 and ρ_α_ = 2/2π = π^–1^ is the angular number density. In this case, *N* and *N*′ are located between the spheres of radius *r* and *r* + *dr*, and the angles α and α + *d*α.

## 3 Results and Discussion

### 3.1 Adjustment of Effective Potentials

Potentials of mean force were used as a first approximation for the interaction between the nucleotides that make up six different high molecular weight structures with varying numbers of nucleotides. The selected structures are a 16S rRNA (rRNA) (PDB ID: 1I94, 1514 nt)^36^ and a 2912-mer (PDB ID: 4V8D, 2912 nt)^37^ from *Thermus thermophilus*; a 16S rRNA (PDB ID: 6O9K, 1539 nt)^38^ and a 23S rRNA (PDB ID: 6O9J, 2840 nt)^38^ from *Escherichia coli*; a 16S rRNA from *Staphylococcus aureus* (PDB ID: 5LI0, 1547 nt);^39^ and a 25S rRNA from *Saccharomyces cerevisiae* (PDB ID: 5TGM, 3149 nt).^40^ The condition met by these structures is that all of the constituent nucleotides must be reported in the PDB files.

The PMF approximation from eq 4 does not reproduce the distribution functions in dense systems; therefore, it is necessary to modify the potentials obtained this way. To improve this first approximation, we used Iterative Boltzmann Inversion (IBI).^41^ In this method, the (*i* + 1)-th approximation of the potential *U*(ξ) can be obtained from

12 $$\beta U_{i+1}\left(\xi \right)=\beta U_{i}\left(\xi \right)+\ln\left[\frac{g_{i}\left(\xi \right)}{g\left(\xi \right)}\right]$$

where *g*_*i*_(ξ) is the distribution function obtained in the *i*-th iteration, while *g*(ξ) is the target function. This algorithm is applied to all interaction potentials.

Simulations were performed using a modification of the Ermak–McCammon algorithm,^21^ the second-order algorithm by Iniesta and García de la Torre for interacting Brownian particles in the overdamped limit,^22^ without considering explicit hydrodynamic interactions. Given the position **r** ^0^ of a particle, the auxiliary position at a subsequent time Δ*t* is given by

13 $$r^{\prime }=r^{0}+\beta \Delta tDF^{0}+R\left(\Delta t\right)$$

where *D* is the self-diffusion coefficient, **F** ^0^ = – ∇*U* is the force exerted on the particle at position **r** ^0^, and **R** is a random displacement with a Gaussian distribution, with zero mean, satisfying $\left\langle R_{i}\left(\Delta t\right)\cdot R_{j}\left(\Delta t\right)\right\rangle =6D\Delta t\delta_{ij}$ , where δ_*ij*_ is the Kronecker delta. Subsequently, the step is repeated but taking the average of the force **F** at positions **r** ^0^ and **r**′:

14 $$r=r^{0}+\beta \Delta tD\left(\frac{F^{0}+F^{\prime }}{2}\right)+R\left(\Delta t\right)$$

For each optimization process of the fitting parameters, 12 simulations were performed for each molecule, where each simulation consisted of 10^5^ thermalization steps, followed by 10^7^ production steps. The reduced time used in the simulations was Δt* = 1.5 × 10^–4^. These values represent a 50% increase in the integration step and a 400% increase in the number of integration steps compared to a previous work.^26^ Figure 5 shows the radial-angular distribution functions, with α = *e*_μν_, obtained from the last iteration of the IBI for Watson–Crick and wobble pairs, along with the distribution of experimental values. The distribution function maxima for GC, AU, and GU are located at 11.4 ± 0.1, 11.6 ± 0.1, and 11.2 ± 0.1 Å, respectively. The values of these maxima are 27.4 ± 8.8 for GC, 14.9 ± 5.6 for AU, and 7.0 ± 2.7 for GU. The inset of Figure 5 presents the final version of the radial-angular potential for the GC interaction. The final values of all fitting parameters can be found in the Supporting Information Section.

**Figure 5:**
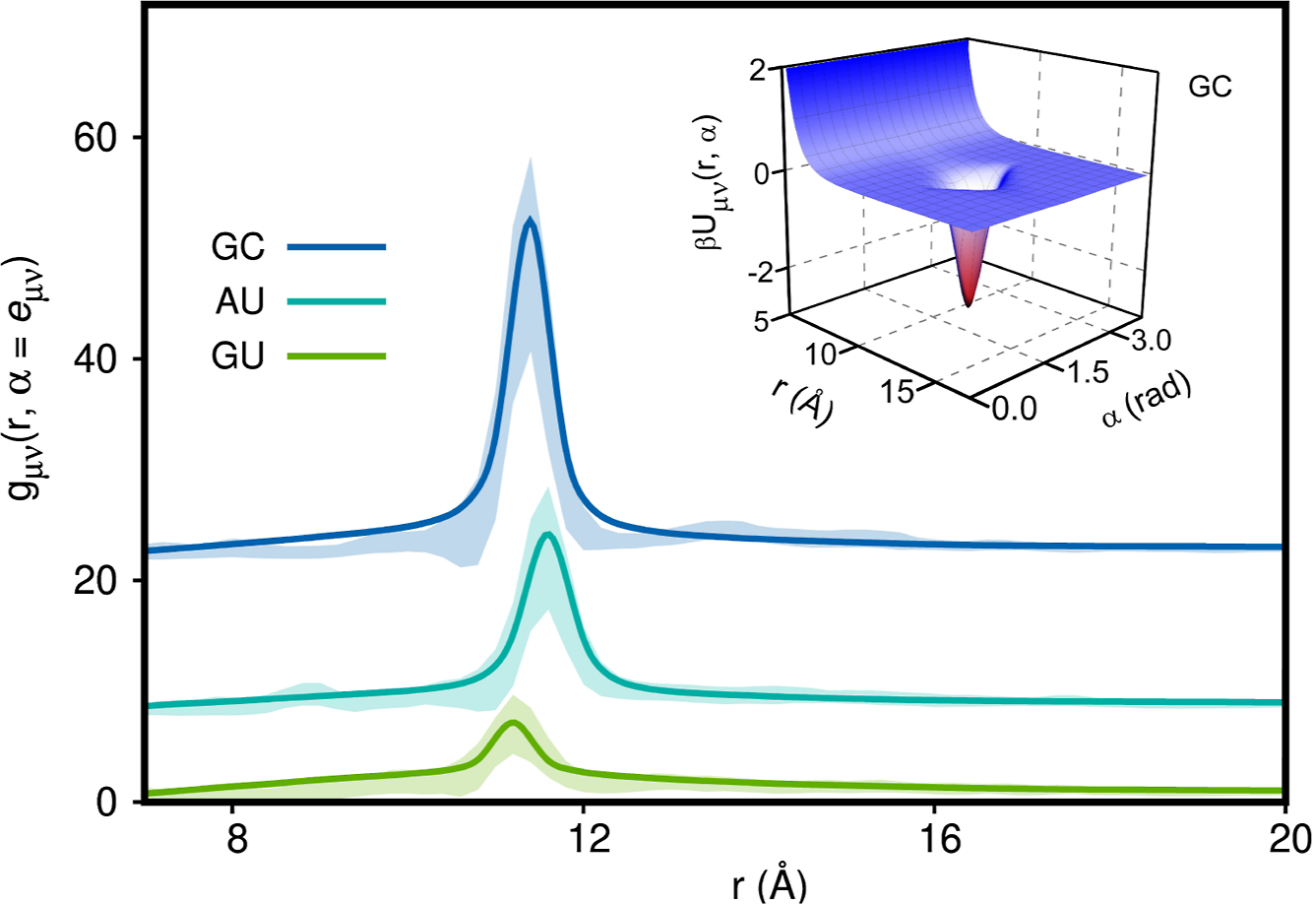
Radial-angular distribution functions of Watson–Crick and wobble pairs with α = *e*_μν_. The lines represent the average of the simulation results, while the light-shaded region represents the distribution of experimental values. For greater clarity, the AU and GC functions have been offset by 8 and 22 units, respectively. Inset: Radial-angular effective potential of the GC pair.

### 3.2 Mechanical Unfolding of RNA Hairpins

All-atom MD simulations have been employed to study the mechanical unfolding of DNA and RNA chains. However, due to the inherent scales of this description, the loading rates utilized are 11 to 12 orders of magnitude higher than their experimental counterparts.^42–44^ When using coarse-grained descriptions, quantities such as the scaling factor from time *t* to dimensionless time t* lack a trivial mapping. To obtain the scaling factor value from the dimensionless time interval Δt* to the time interval with units Δ*t*, the same process reported in previous work was followed.^26^ The temporal calibration process is based on the mechanical unfolding data of the P5ab, P5abcΔA, and P5abc hairpins reported in ref^10^, which represents one of the most comprehensive studies regarding this type of experiments. The chains are parts of the P5 section of *Tetrahymena thermophila*: 1) P5ab with 49 nt (5′-CCGUUCAGUACCAAGUCUCAGGGGAAACUUUGAGAUGGGGUGCUGACGG-3′), 2) P5abcΔ*A* with 64 nt (5′-CCGUUCAGUACCAAGUCUCAGGGGAAACUUUGAGAUGGCCUUGCAAAGGGUAUGGUGCUGACGG-3′), and 3) P5abc with 69 nt (5′-CCGUUCAGUACCAAGUCUCAGGGGAAACUUUGAGAUGGCCUUGCAAAGGGUAUGGUAAUAAGCUGACGG-3′). The three-dimensional configurations of these molecules were obtained from PDB ID: 7XSN.^45^ To ensure that all coupling angles were well-defined, an extra adenine was added to each end of the simulated chain. Mechanical unfolding was simulated at a constant velocity^46^ by coupling a dummy particle to the 3′ end of the chain via a harmonic potential, $U_{s}=\frac{k_{s}}{2}{\left(r-vt\right)}^{2}$ , where *k*_s_ is the spring constant modeling the optical trap, **r** is the distance between the dummy particle and the last nucleotide at the 3′ end, and **v** is the velocity of the dummy particle. The nucleotide at the 5′ end of the chain remains at rest, simulating the action of a micropipette. The dimensionless values for the spring constant and speed must be such that they replicate the behavior of the plateau slope in the FECs and ensure the harmonic potential is ∼10^–3^ the value of the Watson–Crick potential.^47^ The obtained dimensionless values are *k*_s_^*^ = 4.4 × 10^–3^ and v* = 6.9 × 10^–3^, yielding an approximate dimensionless loading rate of *r*_f_^*^≈*k*_s_^*^ *v* ^*^ = 3 × 10^–5^.^16^ Considering that these simulations correspond to experiments performed at a loading rate of *r*_f_ = 10 pN/s,^10^ the spring constant and speed are *k*_s_ = 0.18 pN/nm and ν = 55.6 nm/s, respectively. These values are similar to those previously obtained.^26^

Forty mechanical unfolding simulations were performed for each molecule to obtain the mean value of dimensionless force F*. These dimensionless forces were fitted using least-squares against their experimental counterpart, *F*_exp_. The experimental rupture force values considered are the average of those conducted in EDTA and Mg^2+^, provided that the model was obtained under varied solvent conditions. The experimental results for the mean rupture force, the average dimensionless force obtained from the simulations, and their standard deviations are shown in Table 1. Additionally, the simulated mean rupture force values are shown with their respective unit.

**Table 1:** Experimental (exp) and Simulation-Derived (sim) Values of the Mean Force Required to Unfold the P5ab, P5abcΔA, and P5abc Chains

molecule	length	*F*_exp_ (pN)^10^	F*	*F*_sim_ (pN)
P5ab	49	13.9 ± 0.8	0.36 ± 0.07	13.9 ± 2.9
P5abcΔA	64	12.1 ± 0.9	0.33 ± 0.07	12.9 ± 2.7
P5abc	69	12.0 ± 4.2	0.29 ± 0.07	11.2 ± 2.7

From the performed BD simulations, we obtained an average plateau length of 17.1 ± 3.2 nm for P5ab, 21.8 ± 3.0 nm for P5abcΔA, and 25.3 ± 3.1 nm for P5abc. These values are similar to the experimental results reported by Liphardt,^10^ which were 18 ± 2 nm for P5ab, 21 ± 2 nm for P5abcΔA, and 26 ± 3 nm para P5abc. From these values and the forces in Table 1, we can estimate the Helmholtz free energy as Δ*A* = *F*_sim_Δ*x*, obtaining 141.2 ± 29.1 kJ/mol for P5ab, 169.0 ± 41.7 kJ/mol for P5abcΔA, and 172.2 ± 49.4 kJ/mol for P5abc.

The calibration described in the preceding paragraphs was also validated against experimental data for the P5abc domain unfolding in the absence of Mg^2+^ ions.^11^ This result is presented in Figure 6, which also includes the unfolding curve obtained using the previous version of our model.^26^ In both models, a loading rate of *r*_f_ = 52 pN/s with a spring constant *k*_s_ = 0.47 pN/nm and a speed ν = 111.2 nm/s were applied, matching the experimental conditions. To compensate for potential experimental artifacts from the handles, the simulation-derived curves were adjusted to align with the onset of the experimental unfolding. As shown in the figure, the current model demonstrates better agreement with the experimental unfolding—in terms of both unfolding length and slope—compared to the previous version. This improvement is attributed to a more accurate modeling of the radial-angular potential in the present model. Unlike our previous work, this new version of the model introduces an enhanced definition of the coupling angle α, defined in eq 6, which allows for the correct recovery of the pair distribution function *g*_μν_(*r*, α) across the full range of angles and distances. The difference between the two approaches is illustrated in the inset of Figure 6, which displays a cross-section of the maximum radial-angular distribution function for the AU base pair for both models, compared with their experimental counterpart.

**Figure 6:**
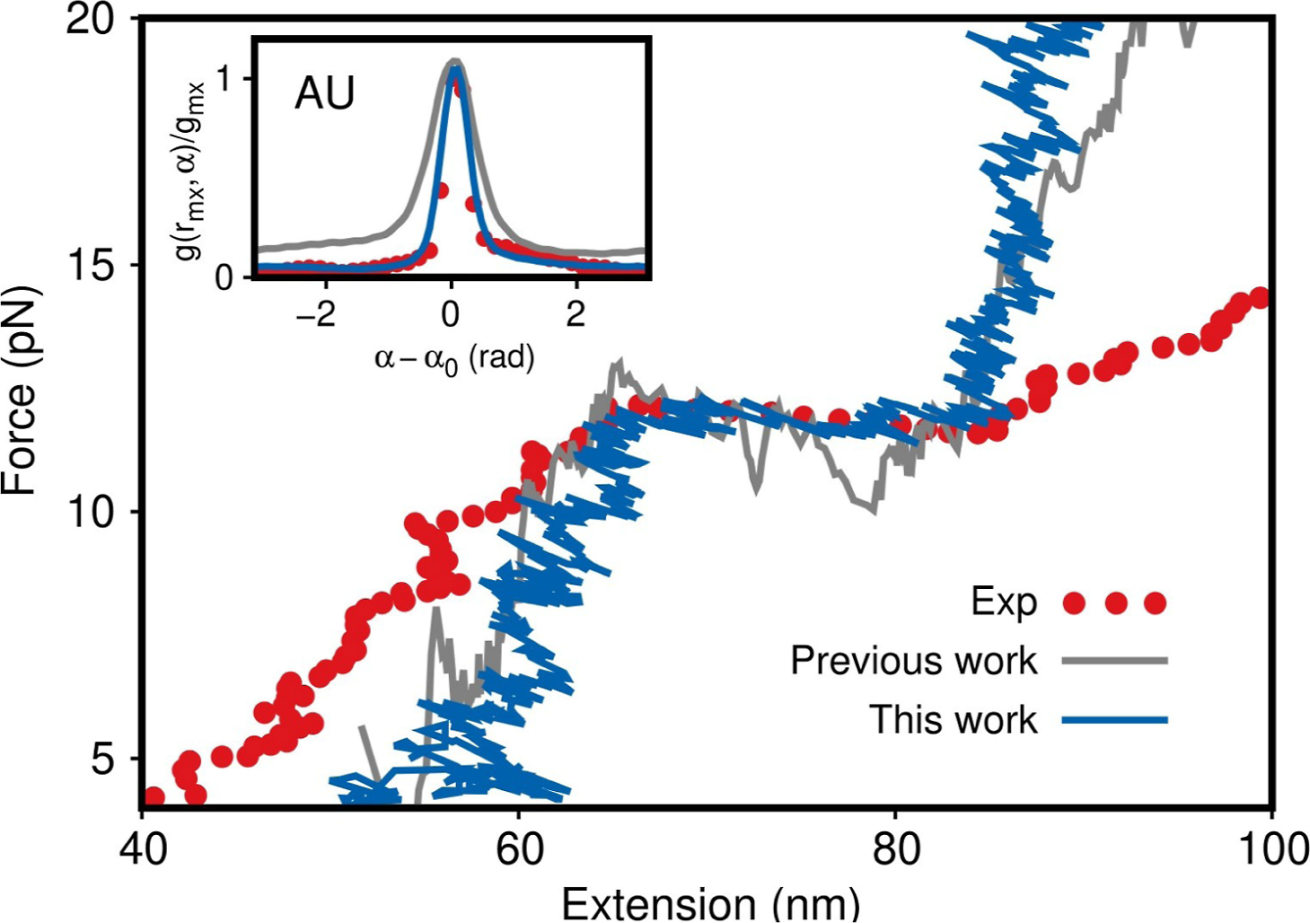
Mechanical unfolding of the P5abc hairpin in the absence of Mg^2+^ ions, referencing the experimental data reported by Liphardt et al.^11^ (red circles) obtained at a loading rate of *r*_f_ = 52 pN/s. The spring constant and the speed were *k*_s_ = 0.47 pN/nm and ν = 111.2 nm/s, respectively. The experimental curve is compared with the FECs obtained at the same loading rate using the model from our previous work^26^ (gray line) and the current study (blue line). Inset: Cross-section of the radial-angular distribution functions for AU bases, with the radial position fixed at its maximum value *r* = *r*_mx_ and α_0_ being the angle at this value. The distribution functions were normalized by their respective maximum values *g*_mx_ (same colors as in the main figure).

Our model was also validated using a hairpin structure that does not belong to the P5 region of *T. thermophila*. This structure consists of a 32 nt chain, synthesized and designated as rHP, which features the following sequence:^48^ 5′-UAGAGAGAGAAAGUUUCGACUUUCUCUCUCUA-3′ and whose structure has not been reported in the PDB. For our purposes, the 3D structure was obtained utilizing AlphaFold 3,^49^ and its unfolding was simulated using the following parameters: *k*_s_ = 0.07 pN/nm (spring constant), ν = 100 nm/s (speed), and *r*_f_ = 7 pN/s (loading rate), mirroring the conditions under which the corresponding experiments were performed.^48^ Figure S5 in the Supporting Information Section presents the comparison between the experimental and simulated mechanical unfolding curves. Yang et al. reported an unfolding force of ∼17 pN and a plateau extension of ∼12.5 nm in force-ramp experiments.^48^ In our simulations, we obtained an average unfolding force of 15.1 ± 3.6 pN and a plateau of 12.1 ± 1.9 nm.

For the following cases, the progression of the unfolding process was monitored by means of a relevant structural property. During such process, we followed the configurational temperature (CT), *T*_conf_, of the molecules through the expression^50^

15 $$k_{B}T_{conf}=-\frac{\left\langle \sum_{i=1}^{N}F_{i}^{2}\right\rangle }{\left\langle \sum_{i=1}^{N}\nabla_{i}\cdot F_{i}\right\rangle }$$

where **F**_*i*_ is the total force exerted on the *i*-th particle and the angular brackets represent an ensemble average. In our case, since each unfolding event is independent, we consider the average over a large number of integration steps of the equations of motion.

Figure 7b shows the FEC for the P5ab molecule. The force required to initiate unfolding is ∼14.4 pN, while the plateau length is ∼15.4 nm. The inset of this figure presents two configurations of the molecule in its folded and unfolded states. Figure 7a displays the CT for the different degrees of freedom considered in the model, as well as the total CT. As previously mentioned, although the calculation of *T*_conf_ involves ensemble averages, given that each unfolding is independent, we calculated the CT over a long time interval, specifically every 1.07 s.

**Figure 7:**
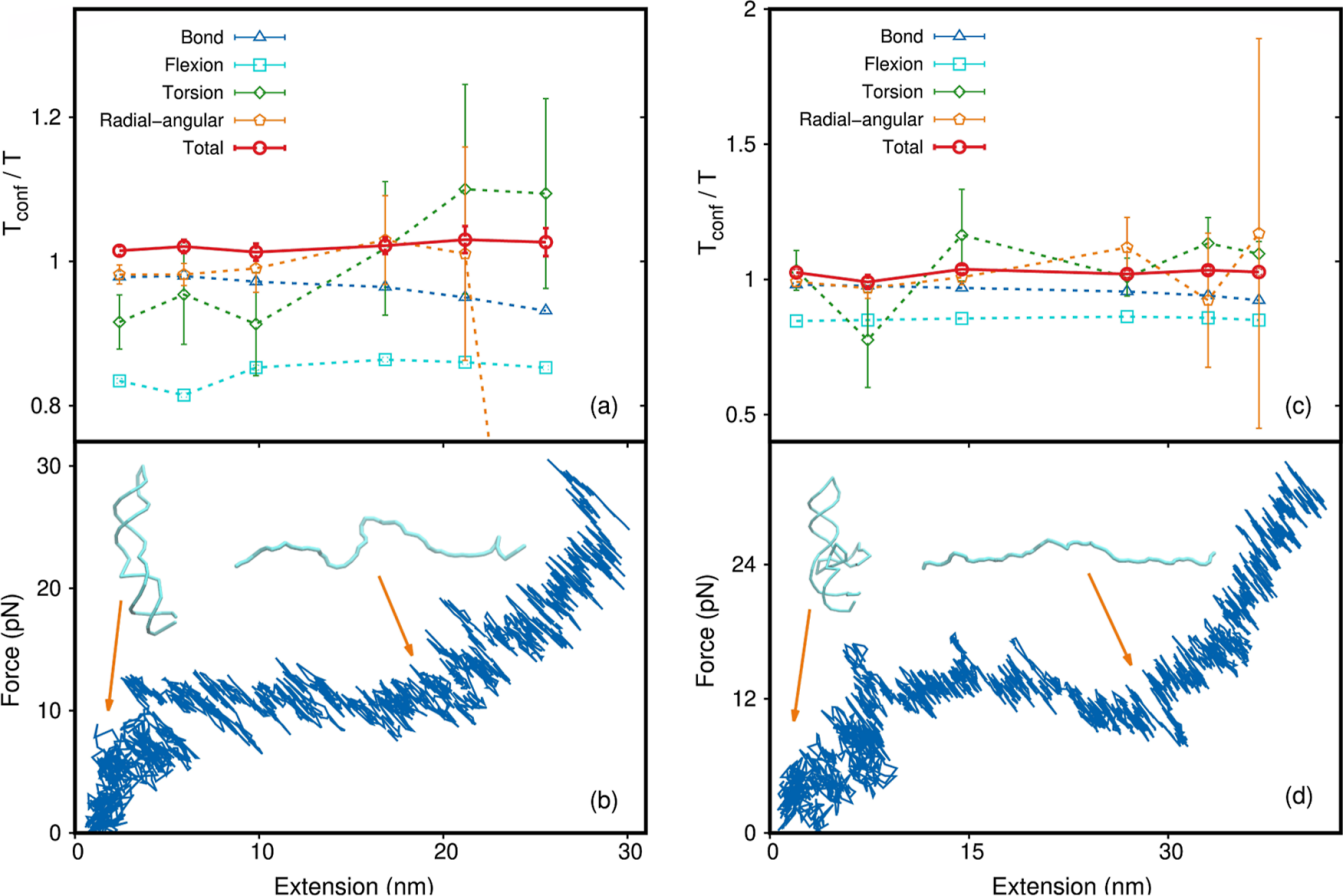
Mechanical unfolding of P5ab and P5abc hairpins. (a, c) CT of P5ab and P5abc during the mechanical unfolding process. The configurational temperatures for the different degrees of freedom of the model and the total CT are shown. (b, d) Two examples of FECs for P5ab and P5abc. Simulations were carried out using a spring constant *k*_s_ = 0.18 pN/nm and a speed ν = 55.6 nm/s, resulting in a loading rate *r*_f_ = 10 pN/s. Data were collected every 2.92 ms. Insets (b, d): tertiary structure of the molecules in the coarse-grained representation (one interaction site per nucleotide) in folded and unfolded states.

During the unfolding process, the CT for the bond contribution decreases monotonically, with an average value of *T*_conf_/*T* = 0.96 ± 0.02. The bending contribution has the lowest value among all contributions and increases slightly, averaging *T*_conf_/*T* = 0.85 ± 0.02, while the torsion and radial-angular terms increase as the chain unfolds, averaging *T*_conf_/*T* = 1.00 ± 0.08 and *T*_conf_/*T* = 0.86 ± 0.35, respectively. Finally, a considerable reduction is observed in the last point of the radial-angular CT where the molecule is fully unfolded, reaching a value of *T*_conf_/*T* ∼ 0.14. This remarkably low value for the radial-angular term is attributed to large fluctuations in both the forces and their divergences. These fluctuations result from the very narrow potential wells of the interactions involved, as illustrated in the inset of Figure 5. For this specific point, the respective numerical values for the numerator and denominator of eq 15 are 7855.3 and −56395.1, after the addition from 10^8^ integration steps. Throughout the unfolding, the total CT remains nearly constant at *T*_conf_/*T* = 1.02 ± 0.01.

Figure 7d shows an example of the FEC for P5abc. The force required to begin unfolding is ∼18.7 pN, with a plateau length of ∼25.9 nm. The inset shows the folded and unfolded states. Figure 7c displays the CT for the various degrees of freedom and the total temperature, calculated every 1.61 s. As with P5ab, the bond term temperature decreases monotonically (*T*_conf_/*T* = 0.96 ± 0.02). Similarly, the bending contribution is the lowest on average (*T*_conf_/*T* = 0.85 ± 0.01), being slightly more stable than in P5ab. In the torsion and radial-angular contributions, oscillations are larger than in previous degrees of freedom, averaging *T*_conf_/*T* = 1.04 ± 0.14 and *T*_conf_/*T* = 1.03 ± 0.09, respectively. In this case, the final radial-angular temperature value does not drop abruptly, though its variation remains significant. The total CT remains nearly constant at *T*_conf_/*T* = 1.02 ± 0.02.

Although the previous model was successful in simulating the unfolding of simple molecules like P5ab and P5abc,^26^ it underestimated interactions in more complex structures such as pseudoknots. To address this, we simulated the mechanical unfolding of two pseudoknots at loading rates similar to those reported in experiments.^13^ The selected structures are an RNA pseudoknot that causes efficient −1 frameshifting in mouse mammary tumor virus, MMTV (PDB ID: 1RNK; 5′-GGCGCAGUGGGCUAGCGCCACUCAAAAGGCCCAU-3′),^51^ and a pseudoknot from the bacteriophage T2 gene 32, PT2G32 (PDB ID: 2TPK; 5′-GCUGACCAGCUAUGAGGUCAUACAUCGUCAUAGCAC-3′).^52^

In the mechanical unfolding experiments of pseudoknots using optical tweezers, Ritchie et al. report pulling rates in a range of 110–270 nm/s and trap stiffnesses of 0.58 and 0.43 pN/nm.^13^ For this reason, the unfolding simulations of PT2G32 and MMTV were performed with an intermediate optical trap stiffness and speed, with values of *k*_s_ = 0.50 pN/nm and ν = 190 nm/s, resulting in a loading rate of *r*_f_ = 95.9 pN/s. For PT2G32, the mean plateau length in the simulations was 13.0 ± 2.3 nm, in excellent agreement with the experiment, which reports Δ*L*_c_ = 13.0 ± 0.6 nm (Table S2 of the supplementary content). On the other hand, the mean force to initiate unfolding obtained from the simulations is *F* = 21.6 ± 4.3 pN. This force is lower than that reported in the experiment, *F*_exp_ = 40 ± 2 pN, while it is similar to what was reported in a previous work.^26^ In the case of MMTV, the experimental result for the plateau length is Δ*L*_c_ = 15 ± 1 nm, while a slightly lower value of 13.9 ± 2.4 nm is obtained in the simulations. Experimentally, the mean force to initiate unfolding has been reported as *F*_exp_ = 26 ± 3 pN, whereas in the simulations, we obtain *F* = 22.0 ± 3.8 pN.

For both pseudoknots, the force required to initiate unfolding is lower than that reported experimentally. However, explicitly including Mg^2+^ ions^28^ yields better agreement for both systems. For the MMTV pseudoknot, the mean unfolding force shifted from 22.0 ± 3.8 pN to 23.5 ± 4.3 pN, a value closer to the experimental result of 26 ± 3 pN reported by Ritchie et al.,^13^ although the mean plateau length decreased to 10.6 ± 2.9 nm (previously 13.9 ± 2.4 nm); the experimentally reported value is 15 ± 1 nm. In the case of the PT2G32 chain, we obtained a mean force of 37.6 ± 7.7 pN (exp. 40 ± 2 pN) and a mean plateau length of 12.7 ± 2.3 nm (exp. 13.0 ± 0.6 nm). The plots corresponding to these results are reported in Figures S6 and S7 of the Supporting Information Section.

Figure 8b shows the unfolding process of PT2G32 without explicit magnesium ions. The force required to initiate unfolding is ∼27.5 pN, while the plateau length is ∼10.3 nm. Figure 8a shows the results of the CT during unfolding. The CT of the bond potential behaves similarly to that of the hairpins, decreasing monotonically with an average value of *T*_conf_/*T* = 0.96 ± 0.02. The CT of the bending potential remains practically constant with a value lower than unity, *T*_conf_/*T* = 0.86 ± 0.01. In this case, the CT of the torsion potential exhibits more variations than its counterpart in the hairpins, with average values of *T*_conf_/*T* = 0.92 ± 0.07. The largest variations in CT again occur in the radial-angular potential, where the highest value obtained is 2.99, located at the last point of the graph. As previously explained, this high value also stems from significant fluctuations in the forces and their divergences. In this particular case, the accumulated values characterizing eq 15 were found to be 934.1 (numerator) and −312.5 (denominator), after 1.8 × 10^8^ time steps. Throughout the simulation, its average is *T*_conf_/*T* = 1.35 ± 0.80. Finally, the total CT, despite oscillating more than in the hairpins, shows stable behavior with an average of *T*_conf_/*T* = 1.00 ± 0.01.

**Figure 8:**
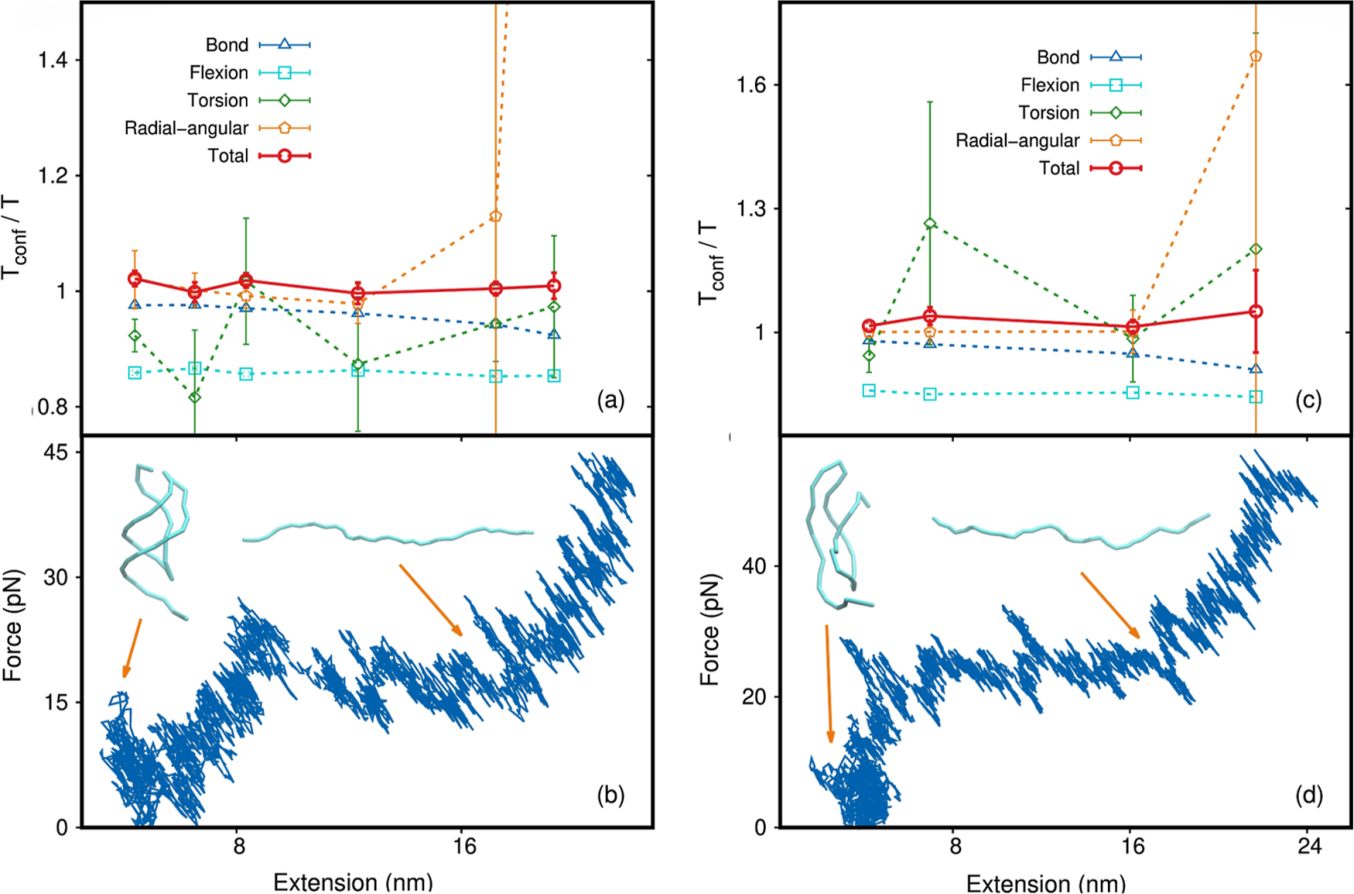
Mechanical unfolding of PT2G32 and MMTV pseudoknots. (a, c) CT of PT2G32 and MMTV during the mechanical unfolding process. The configurational temperatures for the different degrees of freedom of the model and the total CT are shown. (b, d) Two examples of FECs for PT2G32 and MMTV. The simulations were carried out using a spring constant *k*_s_ = 0.50 pN/nm and a speed ν = 190 nm/s, resulting in a loading rate *r*_f_ = 95.9 pN/s. Data were collected every 292 μs for PT2G32 and 598 μs for MMTV. Insets: tertiary structure of the molecules in the one-interacting-site-per-nucleotide representation in folded and unfolded states.

Figure 8d presents the FEC of the MMTV pseudoknot with no explicit Mg^2+^ ions. The force required to begin unfolding is ∼29.3 pN, and the length of rip is ∼16.1 nm. Unlike the previous FECs, the first part of the curve indicates that the end-to-end extension of the chain remains practically constant at ∼4 nm. In this section, it is difficult to calculate different values for the CT; therefore, Figure 8c only contains 4 points during the entire unfolding process. The CT of the bond potential maintains its decreasing behavior with an average of *T*_conf_/*T* = 0.95 ± 0.03. Similar to the bond term, the CT of the bending potential decreases, though not constantly, over time with an average of *T*_conf_/*T* = 0.85 ± 0.01. On the other hand, the torsion potential oscillates throughout the simulation, with an average of *T*_conf_/*T* = 1.10 ± 0.16. In the case of the radial-angular potential, the first three points maintain a value of *T*_conf_/*T* ∼ 1.0. However, the last point increases to ∼1.7. On average, the CT of this degree of freedom is *T*_conf_/*T* = 1.17 ± 0.33. Finally, among the simulations of all structures, the total CT of MMTV is the one that oscillates the most. This can be seen in the lengths of the error bars of the red points in Figure 8a, with a value of *T*_conf_/*T* = 1.03 ± 0.02. This oscillatory behavior could be due to the fact that, like PT2G32, it has the highest loading rate value and, more importantly, MMTV is the shortest structure simulated.

### 3.3 Calculation of Thermodynamic Properties in Mechanical Unfolding

For molecules exhibiting two-stage unfolding (folded-unfolded), we can obtain the work required to unfold the chain from the simulations by calculating the area under the FEC, *W* = ∫**F**·*d* **r**, where **F** is the force exerted by the dummy particle and **r** is the direction in which the force is applied. This work is related to the change in free energy Δ*A* according to the Jarzynski equality:^14^

16 $$\left\langle e^{-\beta W}\right\rangle =e^{-\beta \Delta A}$$

Here, the angle brackets represent an average over many repetitions of the unfolding process. This result is independent of the unfolding trajectory.

In BD simulations, by solving the Langevin equations using the Ermak–McCammon algorithm,^21^ or that of Iniesta and García de la Torre,^22^ while keeping the number of particles, volume, and temperature constant (NVT), the calculated change in free energy will be the Helmholtz free energy Δ*A*. In contrast, in experiments, the quantified change in free energy is the Gibbs free energy, Δ*G*. For quasi-static processes, we can relate the change in these two energies and the applied external work:^23^ Δ*G* = Δ*A* + *W*.

One method used to calculate the free energy change in experiments is calculating the product of the average force required to initiate unfolding and the average plateau length Δ*G* = *F*_1/2_Δ*x*_1/2_,^10^ where *F*_1/2_ is the average force applied to start mechanical unfolding and Δ*x*_1/2_ is the average length of the plateau in the FECs. This process is valid when the system is near equilibrium. For this reason, this procedure is not valid for obtaining the free energy in pseudoknot unfolding, as their loading rate is an order of magnitude higher than that used for hairpins. Despite being a good approximation, this product differs from the calculation of the area under the FECs for several reasons, such as the presence of intermediate states or a nonzero plateau slope. However, theoretical models are also applied to estimate the value of Δ*G*.^13,53^

From BD simulations, we calculated the work *W* from the FECs. The Jarzynski estimator for the free energy change,^14^ $\Delta A=-k_{B}T\;\ln\left\langle e^{-\beta W}\right\rangle$ , and the Gibbs free energy change Δ*G* were also calculated. At this point, we highlight the importance of the Jarzynski estimator being able to converge within a finite number of unfolding trajectories. Figure S4 in the Supporting Information Section shows an example of the convergence of this estimator, for the molecule P5abc, on a curve obtained for different trajectories, which is presented in comparison with the curve derived from the second-order cumulant expansion.^11^ Similar results were obtained for some other RNA molecules. The work and free energy changes are shown in Table 2 and contrasted with the free energy changes obtained from mechanical unfolding experiments, Δ*G*_exp_. Furthermore, the free energy change using the RNAstructure Web Server, Δ*G*_theo_,^54^ is reported. Additionally, the free energy changes for the pseudoknots, calculated using Turner’s free energy rule,^56,57^ are included. For PT2G32, the free energy values for the constituent helical stems are 41.8 and 51.9 kJ/mol, totaling 93.7 kJ/mol. Meanwhile, for MMTV, Δ*G* values of 50.6 and 34.7 kJ/mol have been reported for its two helices, giving a total of 85.3 kJ/mol.^55^

**Table 2:** Values Obtained from the Simulations: Work Obtained from the Integrals of the FECs *W*, Change in Helmholtz Free Energy Δ*A*, and Change in Gibbs Free Energy Δ*G* for the Simulated Molecules at Loading Rates Similar to the Experimental Ones; Change in Gibbs Free Energy in Mechanical Unfolding Experiments, Applying the Jarzynski Equality Δ*G*_exp_, and Theoretical Change in Free Energy Δ*G*_theo_ Using the RNAstructure Method^54^ and Turner’s Free Energy Rule,^56,57^ in Parentheses

molecule	*W* (kJ/mol)	Δ*A* (kJ/mol)	Δ*G* (kJ/mol)	Δ*G*_exp_ (kJ/mol)	Δ*G*_theo_ (kJ/mol)
P5ab	94.1 ± 4.9	52.9 ± 2.2	147.0 ± 7.1	150.5 ± 9.2 ^10^	158.2
P5abcΔA	128.7 ± 5.4	45.4 ± 2.4	174.1 ± 8.1	156.5 ± 17.7 ^10^	168.2
P5abc	152.5 ± 6.6	77.3 ± 1.5	229.8 ± 8.1	172.3 ± 44.3 ^10^	141.0
PT2G32	133.3 ± 7.2	41.1 ± 2.5	174.4 ± 9.7	141.2 ± 4.3 ^13^	64.9 (93.7)
MMTV	145.2 ± 8.1	62.1 ± 2.4	207.3 ± 10.5	153.9 ± 3.5 ^13^	79.9 (85.3)

In general, it can be observed that the Δ*G*_exp_ and Δ*G* values for the hairpins are in good agreement. Similarly, Δ*G* and Δ*G*_theo_ are comparable, except in the case of P5abc. In mechanical unfolding experiments, the large variation in the free energy of P5abc is due to the fact that experiments report rupture forces ranging from 7 to 22 pN and rip lengths from 13 to 26 nm.

The free energies for the pseudoknots were obtained from the curves reported by Ritchie et al.^13^ In the case of MMTV, the authors report unfolding starting from an alternative structure; if this unfolding is taken into account, Δ*G* is reduced to 75.3 ± 4.3 kJ/mol. In this case, the calculation of the pseudoknots’ free energy was performed considering that the thermodynamic stabilities of the isolated helices determine the order of the structural transitions.^55^

In the case of pseudoknots, the result furthest from the unfolding experiments is for MMTV, yielding a ratio of Δ*G*/Δ*G*_exp_ ∼1.3, while approximately 1.2 is obtained for PT2G32. On the other hand, the theoretical results show the greatest discrepancy.

For both simple and complex systems, one of the most difficult thermodynamic functions to quantify in numerical simulations is entropy, *S*.^58^ Various methods exist to calculate the configurational entropy of systems such as RNA,^25^ but even in experiments, theoretical models are used to provide an estimate of the entropy change.^10^ In BD simulations of mechanical unfolding, we can calculate the change in entropy Δ*S* when moving from the folded to the unfolded state through the change in internal energy Δ*E* and Δ*A*.

Some of the most commonly used theoretical models to estimate entropy change are the ideal chain (simple random walk),^8^ the self-avoiding walk (SAW),^8^ and the worm-like chain (WLC).^47,59^ The entropy for the ideal chain is given by

17 $$S\left(r\right)=S_{0}-\frac{3k_{B}r^{2}}{2Na^{2}}$$

where *r* is the end-to-end distance and $S\left(0\right)=\frac{3}{2}k_{B}\;\ln\left(\frac{3}{2\pi Na^{2}}\right)$ . The entropy change is given by Δ*S* = 3*k*_B_Δ*x* ^2^/2*Na* ^2^, where we have considered Δ*x* as the length of the unfolding plateau in the FECs (length of rip). The ideal chain is a model with trivial mathematical properties, but the SAW model has complex mathematical properties. In this model, entropy can be estimated by the expression^8^

18 $$S\left(r\right)=S\left(0\right)-k_{B}{\left(\frac{r}{R_{g}}\right)}^{\delta }$$

where δ = (1 – ν)^−1^. Since δ and *R*_g_ depend on ν, the entropy change will be given by

19 $$\Delta S=k_{B}\left[{\left(\frac{r_{i}}{R_{g,i}}\right)}^{\delta_{i}}-{\left(\frac{r_{f}}{R_{g,f}}\right)}^{\delta_{f}}\right]$$

From the analysis of the simulation results, we obtain that the average initial and final values of ν for P5ab are ν_*i*_ = 0.36 ± 0.05 and ν_f_ = 0.62 ± 0.03, respectively. For P5abcΔA, we obtain ν_*i*_ = 0.35 ± 0.04 and ν_f_ = 0.63 ± 0.03, while the values for P5abc are ν_*i*_ = 0.34 ± 0.02 and ν_f_ = 0.64 ± 0.03. In the case of pseudoknots, we obtain ν_*i*_ = 0.33 ± 0.05 and ν_f_ = 0.62 ± 0.03 for PT2G32 and ν_*i*_ = 0.30 ± 0.05 and ν_f_ = 0.62 ± 0.03 for MMTV. In all cases, these Flory exponent values indicate a transition from a collapsed state, where attractive interactions dominate, to an expanded chain. One of the most used models to describe mechanical unfolding experiments is the WLC. In this model, the applied force *f* is given by

20 $$\frac{fl_{p}}{k_{B}T}=\frac{1}{4}{\left(1-\frac{r}{Lc}\right)}^{-2}-\frac{1}{4}+\frac{r}{Lc}$$

When applying this model, it is considered that the entropy change can be obtained from the integral of the force, Δ*S* = −1/*T*∫_0_^Δ*x*^ *f*(*r*)*dr*

21 $$\Delta S=-\frac{k_{B}L_{c}}{4l_{p}}\left[\frac{1}{1-\Delta x/L_{c}}-\frac{\Delta x}{L_{c}}+\frac{2\Delta x^{2}}{L_{c}^{2}}\right]$$

Using Δx* = Δ*x*/*L*_c_, we obtain an entropy change $\Delta S=-\frac{k_{B}L_{c}}{4l_{p}}\left[{\left(1-\Delta x^{*}\right)}^{-1}-\Delta x^{*}+2{\left(\Delta x^{*}\right)}^{2}\right]$ .

From the simulation results, we obtained Δ*A* and Δ*E*, with which we calculated the change in entropy during the unfolding process. The three models that quantify entropy changes depend on the initial and final end-to-end distance (or their difference) and the radius of gyration, meaning that the entropies obtained with these methods are only configurational entropies, while those obtained from the BD simulation, using the Jarzynski equality, are thermodynamic entropies.

The changes in configurational entropies are negative because the number of possible configurations the molecule can have is greater in the folded state than in the unfolded state; thus, *S*_*i*_ = *k*_B_ ln­(Ω_*i*_) > *S*_f_ = *k*_B_ ln­(Ω_f_). On the other hand, we observe that Δ*S* > 0 in the simulations. The product *T*Δ*S* represents the amount of heat the system absorbs for the unfolding process to occur isothermally. Part of this heat is used to break hydrogen bonds, represented by the radial-angular potential.

Table 3 shows the Δ*S* results from the simulations and their comparison with values obtained from theoretical models, considering the *l*_p_ and *L*_c_ values found in the analysis of the set of 1944 RNA molecules obtained from the PDB. From these results, we can observe that the magnitude of the entropy change in the three models is approximately double that obtained in the BD simulations.

**Table 3:** Unfolding Process Length of Rip, Internal Energy Change Δ*E*, Simulation Entropy Change Δ*S*, and Entropy Change of the Gaussian (Ideal) Chain Δ*S*_G_, the Self-avoiding Chain Δ*S*_SAW_, and the WLC Δ*S*_WLC_ for the Simulated Molecules

molecule	Δ*x* (nm)	Δ*E* (kJ/mol)	Δ*S* (*k*_B_)	–Δ*S*_G_ (*k*_B_)	–Δ*S*_SAW_ (*k*_B_)	–Δ*S*_WLC_ (*k*_B_)
P5ab	17.1 ± 3.2	83.9 ± 4.5	12.5 ± 2.7	30.5 ± 1.8	23.9 ± 1.1	25.2 ± 2.7
P5abcΔA	21.8 ± 3.0	89.2 ± 3.7	16.8 ± 1.7	37.3 ± 1.5	27.8 ± 1.3	27.0 ± 2.9
P5abc	25.3 ± 3.2	99.4 ± 3.2	8.9 ± 1.9	46.8 ± 1.7	30.9 ± 1.3	34.3 ± 3.7
PT2G32	13.1 ± 2.3	62.7 ± 4.0	8.7 ± 2.6	24.2 ± 1.3	24.5 ± 1.2	18.4 ± 2.0
MMTV	13.9 ± 2.4	81.8 ± 4.7	7.9 ± 2.9	28.6 ± 1.7	26.1 ± 1.5	28.1 ± 3.0

Regardless of the theoretical model considered, the norm of the entropy change is greater than that obtained from simulations. On average, the ratio |Δ*S*_M_|/Δ*S* = 2.9 ± 0.4, where *M* represents the model M = *G*, *SAW*, or *WLC*. The structure exhibiting the highest value for this ratio is P5abc across all models, while those with the lowest values are P5abcΔ*A* and P5ab, depending on the model. It is worth noting that these analytical models neglect both local (bond, bending, and torsion) and nonlocal (such as Watson–Crick base pairs) degrees of freedom. Specifically, the Gaussian chain model overestimates the −Δ*S*/*k*_B_ term because it neglects excluded volume and attractive interactions. The SAW model is closer to reality in a good solvent; however, it may still overestimate this term as it accounts for neither local rigidity nor attractive interactions that collapse the chain. The WLC model depends on rigidity. When applied with the correct persistence length, it can be highly accurate in describing the conformational entropy of the main chain. When compared to a real chain with additional interactions—such as base pairing—the WLC model alone overestimates the −Δ*S*/*k*_B_ term because the real chain is subject to extra constraints.

Nevertheless, it can be mentioned that all results are of the same order of magnitude, consistent with theoretical models for calculating the configurational entropy of RNA chains.^25,60^ In the Supporting Information of their article, Liphardt et al.^10^ estimate the entropy change using the WLC model for P5ab by employing other values for *l*_p_ and *L*_c_, obtaining a range for −Δ*S*/*k*_B_ between 13.7 and 21.4, with an average value of −Δ*S*/*k*_B_ = 17.7. This value is similar, in absolute value, to those obtained from our simulations.

### 3.4 Predictions of the Mechanical Unfolding of Two Molecules of Medical Interest

The results of the BD simulations for the aforementioned molecules can be compared with their experimental counterparts and show excellent agreement. However, one of the objectives of this model is to perform predictions of the mechanical unfolding of RNA chains without using optical tweezers experiments. In this regard, we have performed unfolding simulations of molecules that are elements of two pandemic pathogens: on one hand, an influenza A virus promoter, consisting of 31 nucleotides (PDB ID: 1JO7; 5′-AGUAGAAACAAGGCUUCGGCCUGCUUUUGCU-3′).^61^ This hairpin is involved in processes that are prerequisites for viral replication. On the other hand, a SARS-CoV-2 frameshift stimulation element, an RNA molecule composed of 88 nucleotides (PDB ID: 6XRZ; 5′-GUUUUUAAAC GGGUUUGCGGUGUAAGUGCAGCCCGUCUUACACCGUGCGGCACAGGCACUAGUACUGAUGUCGUAUACAGGGCUUUUG-3′).^27^ This molecule is of particular interest because it regulates the expression of viral proteins. The simulations were conducted at a loading rate equal to that of the pseudoknots.

The FEC results for 1JO7 show a typical hairpin unfolding, yielding a single plateau that, in some cases, exhibits intermediate states. The average unfolding force obtained is 22.5 ± 4.7 pN, with a rip length of 13.0 ± 2.3 nm. In the case of 6XRZ, simulation results consistently show three plateaux in the FECs. The average forces to initiate the ruptures are 25.4 ± 4.5, 40.8 ± 6.2, and 54.9 ± 4.4 pN, and the plateau lengths are 13.4 ± 4.2, 11.5 ± 4.3, and 12.3 ± 2.8 nm.

Usually, hairpins and pseudoknots transition from a folded to an unfolded state; or, in the case of pseudoknots, they may begin unfolding from an alternative configuration.^13^ However, if the 3D structure of the molecule to be unfolded is more complex, several unfolding pathways may exist. This phenomenon has been previously reported.^12^ For 6XRZ, we found that intermediate states can occur in the first plateau, and on several occasions, we found that the first and second plateaux merge to give way to a single plateau with an extension of ∼20.6 nm and a rupture force of ∼49.0 pN.

Figure 9b shows an example of the FEC for 1JO7. The force required to initiate unfolding is ∼28.7 pN, and the rip length is ∼13.6 nm. Figure 9a presents the configurational temperatures of the different degrees of freedom during unfolding. The configurational bond temperature maintains a downward behavior, with a mean value of *T*_conf_/*T* = 0.95 ± 0.03. The bending potential CT shows a behavior similar to that obtained for P5ab, starting at *T*_conf_/*T*∼ 0.75 and later stabilizing at *T*_conf_/*T* ∼ 0.85, with average values of *T*_conf_/*T* = 0.83 ± 0.05. The CT of the torsion potential reaches a maximum value of *T*_conf_/*T* = 1.4 ± 0.76 during the transition between the unfolding plateau and the fully unfolded state; nevertheless, it maintains an average value of *T*_conf_/*T* = 1.06 ± 0.26 throughout the entire simulation. Again, the CT of the radial-angular term shows very high values in the region where the molecule is fully unfolded, yielding a mean value of *T*_conf_/*T* = 2.91 ± 3.87. Finally, the total CT exhibits oscillating but stable behavior, with a mean of *T*_conf_/*T* = 1.00 ± 0.05. Despite the large oscillations in the torsion and radial-angular contributions, we find that the total CT remains stable throughout the unfolding process. Figure 9d shows the intermediate state occurring in the first plateau at approximately 10 nm of end-to-end distance and an applied force of ∼28.0 pN. Figure 9c displays the CT of 6XRZ during the mechanical unfolding process. The CT associated with the bond potential decreases during the unfolding process, with a mean value of *T*_conf_/*T* = 0.96 ± 0.01. The CT of the bending potential again shows the minimum value, with an average of *T*_conf_/*T* = 0.86 ± 0.005. In this unfolding process, fluctuations in the CT of the torsion term reach the maximum value, with an average of *T*_conf_/*T* = 1.11 ± 0.18, while the radial-angular term remains more stable than in other unfolding events, presenting an average of *T*_conf_/*T* = 0.97 ± 0.08. Notably, the behavior of the total CT is predominantly determined by the torsion and radial-angular terms; that is, their behaviors throughout the unfolding process are similar. Finally, the total CT has an average value of *T*_conf_/*T* = 1.02 ± 0.03.

**Figure 9:**
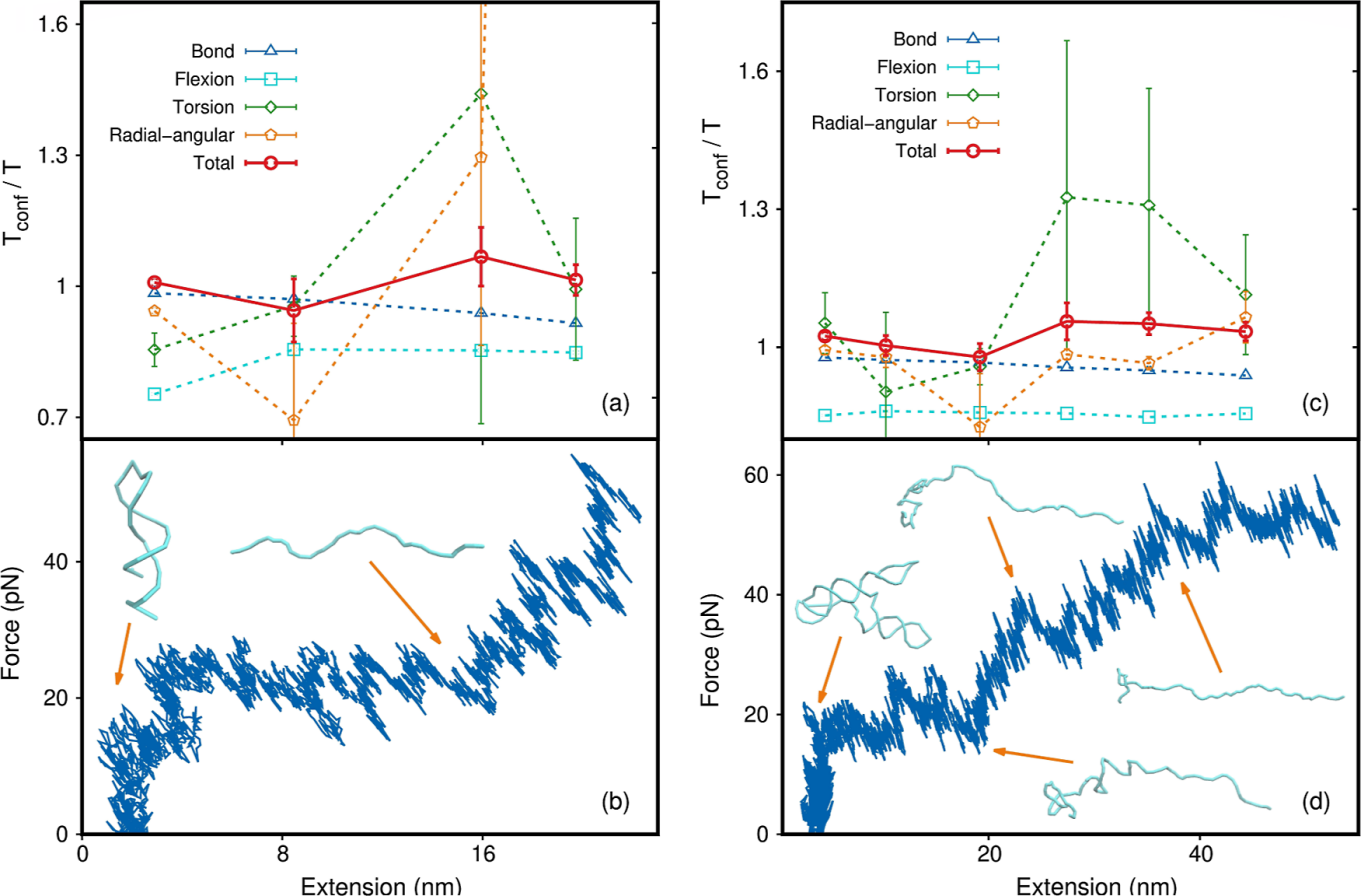
Mechanical unfolding of the 1JO7 and 6XRZ molecules. (a, c) CT of 1JO7 and 6XRZ during the mechanical unfolding process. The configurational temperatures for the different degrees of freedom of the model and the total CT are shown. (b, d) Example of the FEC of 1JO7 and 6XRZ. The simulations were performed using a spring constant *k*_s_ = 0.50 pN/nm and a speed *v* = 190 nm/s, resulting in a loading rate *r*_f_ = 95.9 pN/s. Data were collected every 598 μs. Inset: tertiary structure of the molecules in a one-interacting-site-per-nucleotide representation at different unfolding states.

From the set of simulations, we obtained an average work *W* = 149.0 ± 6.7 kJ/mol for 1JO7 and free energy changes of Δ*A* = 84.7 ± 2.2 kJ/mol and Δ*G* = 233.7 ± 8.9 kJ/mol. Comparing the simulation results with those obtained from the RNAstructure, we find Δ*G*/Δ*G*_theo_ ∼ 3.96, similar to the results for PT2G32 and MMTV, which yield 2.69 and 2.60, respectively. In the experimental structure of 1JO7, an (A–A)·U motif is present between A_10_-U_22_ and A_11_-U_22_. It is noteworthy that the RNAstructure fails to accurately predict this motif, identifying only the A_10_-U_22_ base pair. Furthermore, if this pairing is substituted for A_11_-U_22_, the tool does not provide a value for Δ*G*_theo_.

During mechanical unfolding, the Flory exponent goes from ν_*i*_ = 0.30 ± 0.02 (compact) to ν_f_ = 0.61 ± 0.03 (stretched). The internal energy change obtained for 1JO7 is Δ*E* = 124.9 ± 6.7 kJ/mol, and the entropy change is Δ*S*/*k*_B_ = 16.2 ± 3.6. The Gaussian, SAW, and WLC models yield entropy changes of −Δ*S*/*k*_B_ = 27.7 ± 2.0, 24.8 ± 1.6, and 32.9 ± 3.5, respectively.

Regarding the unfolding of 6XRZ, given that there are several partial unfoldings, calculating the mean work is not useful. In this case, *W* is considered as the area under the FECs from the beginning of the first plateau to the end of the last. This same consideration will be applied to Δ*A* and Δ*G*.

The internal energy change and the average work in the unfolding of 6XRZ are Δ*E* = 260.9 ± 17.8 kJ/mol and *W* = 739.9 ± 52.8 kJ/mol, respectively. Meanwhile, the changes in free energies are Δ*A* = 203.8 ± 12.1 kJ/mol and Δ*G* = 943.7 ± 55.3 kJ/mol. Comparing this last result with that obtained from the RNAstructure, we have Δ*G*/Δ*G*_theo_ ∼ 6.61. This result is presented to complete our analysis, as the tool does not provide a correct secondary structure, despite how efficient it can be with other types of molecules.

Assuming that mechanical unfolding experiments overestimate free energy changes at high loading rates, we can readjust the Δ*G* values for the 1JO7 and 6XRZ predictions by using the average of the previously calculated ratios for the analyzed pseudoknots. Consequently, we obtain Δ*G* values of 88.5 kJ/mol for 1JO7 and 357.5 kJ/mol for 6XRZ. There is currently no definitive evidence regarding the overestimation of Gibbs free energy in unfolding experiments. However, Δ*G* results from experiments at high loading rates are typically contrasted with other theoretical models rather than with the results obtained from the Jarzynski equality. In this regard, further research is required.

During unfolding, the Flory exponent has initial and final values of ν_*i*_ = 0.35 ± 0.04 and ν_f_ = 0.69 ± 0.05. Finally, the entropy change during unfolding is Δ*S*/*k*_B_ = 23.0 ± 8.2. The entropy changes from the Gaussian, SAW, and WLC models for 6XRZ yield −Δ*S*/*k*_B_ = 88.1 ± 8.2, 70.4 ± 7.0, and 43.6 ± 4.6, respectively. Although the WLC model yields results whose absolute value is closest to the simulations, it presents the largest uncertainty because it is highly sensitive to variations in Δ*x*, which in this case Δ*x* changes considerably due to the different unfolding pathways.

## 4 Conclusions

We modified the definition of the coupling angle in our model to correctly control the width of the angular component of the radial-angular potential. Simultaneously, we increased the statistics regarding the number of molecules used to calculate the distribution functions, moving from 501 to 1944 for local interactions and 818 for nonlocal interactions, considering that only RNA structures with at least 1000 nucleotides were used. These molecules range from 3 to 4298 nucleotides. Based on the analysis of the 1944 structures obtained from the PDB, we calculated several characteristic lengths for polymer chains, such as contour length, radius of gyration, and persistence length. Using these lengths alongside the bond distribution function, we estimated the average distance between nucleotides, obtaining *a* = 5.5 ± 0.4 Å. Additionally, we calculated the Flory exponent value, ν = 0.33 ± 0.01, and the average fractal dimension, *d*_F_ = 3.03 ± 0.09. All these values are in excellent agreement with previously reported experimental and theoretical results.

The optimization of the fitting parameters for the effective potentials was performed by using IBI. Our potentials recover the distribution functions of the different degrees of freedom for large RNA molecules, ranging from 1514 to 3149 nucleotides. We simulated the mechanical unfolding of hairpins and pseudoknots under conditions similar to those of experiments. The hairpin unfolding results at 10 pN/s are in excellent agreement with experimental data, while the pseudoknot results at a loading rate of 95.9 pN/s differ slightly from those obtained experimentally. Results for pseudoknots clearly improve by explicitly modeling the interaction with magnesium ions.

During the unfolding simulations, we monitored the CT of the model’s different degrees of freedom as well as the total CT. In the mechanical unfolding of hairpins and pseudoknots, the *T*_conf_/*T* ratio is close to unity; however, in the pseudoknot simulations, oscillations tend to be larger, possibly due to the higher loading rate.

From the resulting FECs, we calculated the Helmholtz free-energy change using the Jarzynski equality. We also quantified the Gibbs free energy change through the integration of the FECs. Along with monitoring the internal energy change in the mechanical unfolding simulations, we calculated the entropy change between the folded and the unfolded states. These results were contrasted with theoretical models that estimate the change in entropy, such as the Gaussian phantom chain, self-avoiding chain, and wormlike chain. The simulated results are of the same order of magnitude as the absolute values of reported theoretical estimates. More importantly, our results already have the correct sign for the entropy change. Finally, given the accuracy and relatively low computational cost of our model, it may serve as a valuable supportive tool in the design of novel messenger-RNA-based vaccines or some other therapies.

## Supplementary Material







## Notes

Two examples of unfolding simulations of the hairpin P5ab (MP4)
Pseudoknot PT2G32 (MP4)
Convergence of the pair distribution functions toward their average value for different local degrees of freedom; convergence of the Jarzynski estimator; FEC of a hairpin (rHP); mechanical unfolding of two pseudoknots (MMTV and PT2G32) in the presence of magnesium ions; and model’s degrees of freedom and parameters (PDF)
