# Supplementary material for: Structural and Thermodynamic Properties of RNA Molecules Using a Knowledge-Based Model
Source: J Chem Theory Comput. 2026 Jul 13;22(14):7116–30. doi: 10.1021/acs.jctc.6c00491 (PMC13420581; doi:10.1021/acs.jctc.6c00491)
Supplement: Supplementary file 3 [file ct6c00491_si_003.pdf]

Supporting Information: Structural and thermodynamic properties of RNA molecules using a knowledge-based model

Mario Villada-Balbuena*,†,‡ and Mauricio D. Carbajal-Tinoco*,†

†*Departamento de Física, Centro de Investigación y de Estudios Avanzados del IPN, Av. Instituto Politécnico Nacional No. 2508, Col. San Pedro Zacatenco, CP 07360 Ciudad de México, Mexico*

‡*Tecnologico de Monterrey, Escuela de Ingeniería y Ciencias, Av. Eugenio Garza Sada 2501, CP 64849, Monterrey, Nuevo León, Mexico*

E-mail: mario.villada@tec.mx; mauricio.carbajal@cinvestav.mx

Abstract

This Supporting Information Section contains supplementary figures, animation descriptions, and the complete set of parameters used in the model.

S1 Unfolding animations

Two examples of unfolding simulations of the hairpin P5ab (mp4) and the pseudoknot PT2G32 (mp4) are included.

S2 Figures

S2.1 Convergence figures

Figures S1-S3 display the convergence of pair distribution functions toward their average value for 3 degrees of freedom. Figure S4 shows the convergence of the Jarzynski estimator.

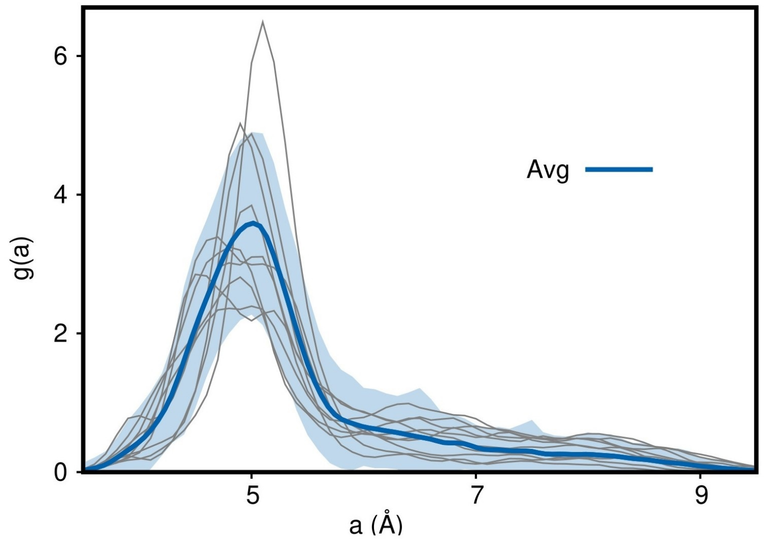

Figure S1: Bond pair distribution functions. Gray lines: pair distribution functions for the ten combinations of nucleotides. Blue line: the average of the ten distribution functions. Light blue bar: error bars of the average.

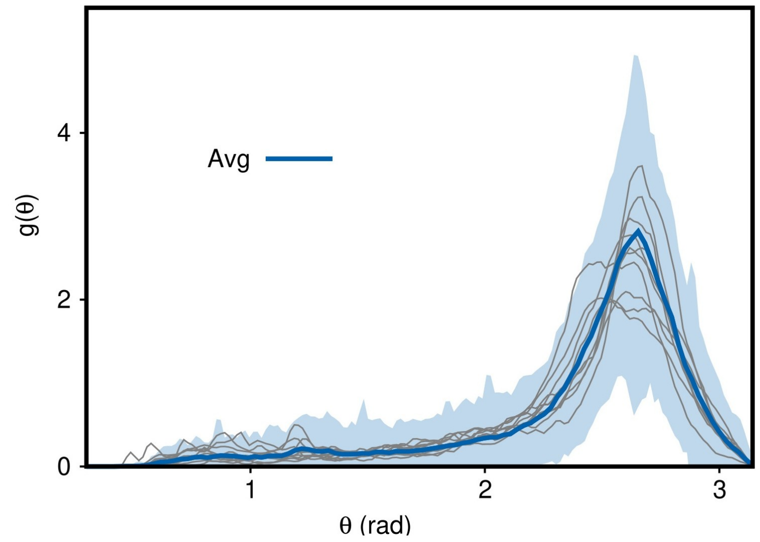

Figure S2: Bending pair distribution functions. *Ibid.* to the preceding figure.

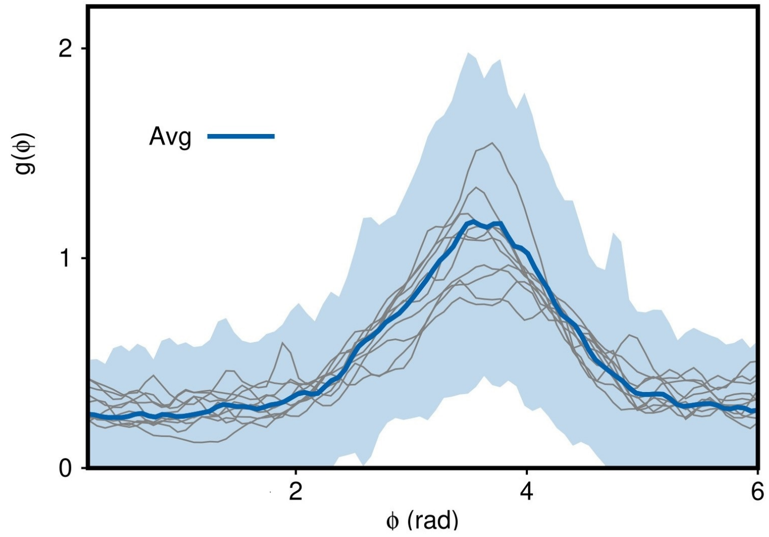

Figure S3: Torsion pair distribution functions. *Ibid.* to the preceding figure.

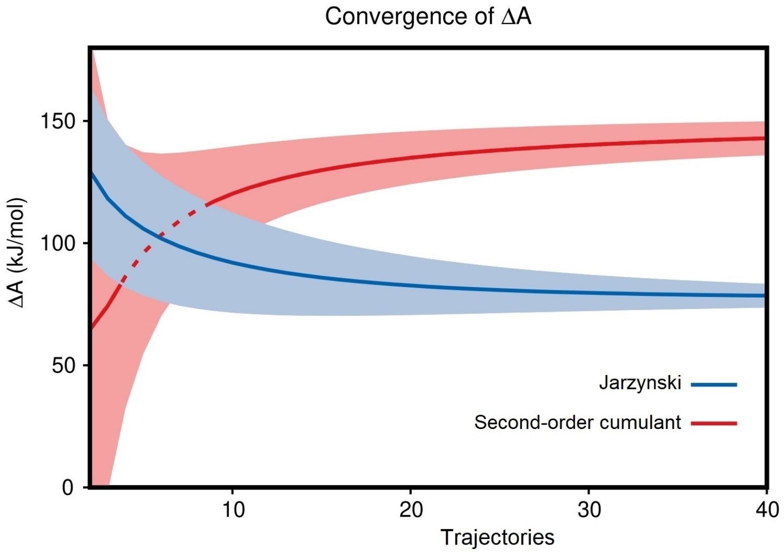

Figure S4: Convergence of the Jarzynski estimator,[14] $\Delta A = -k_{\mathrm{B}}T \ln \left\langle e^{-\beta W} \right\rangle$, as a function of the number of unfolding trajectories for the molecule P5abc (blue line), and shown in comparison with the second-order cumulant approximation,[11] $\Delta A = \langle W \rangle - \beta\sigma^2/2$ (red line), with σ being the standard deviation of the work distribution. Note that both approaches are equivalent only in the case of a Gaussian work distribution.

S2.2 Unfolding figures

Figures S5-S7 present three additional cases of experimental mechanical unfolding compared with the results of our model.

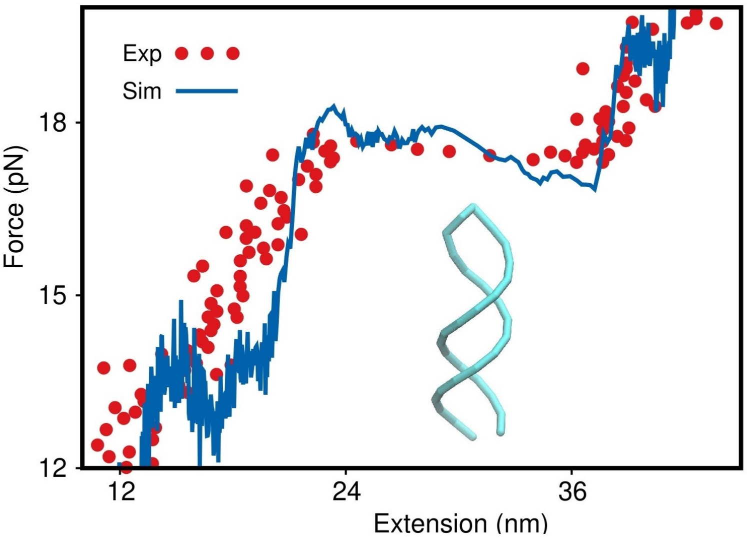

Figure S5: Mechanical unfolding of the rHP hairpin (shown in cyan) obtained from BD simulations carried out with the following parameters: $k_s = 0.07$ pN/nm (spring constant), $v = 100$ nm/s (speed), and $r_f = 7$ pN/s (loading rate). The simulation curve (blue line) is shown in comparison with experimental data performed with the same specifications[48] (red circles).

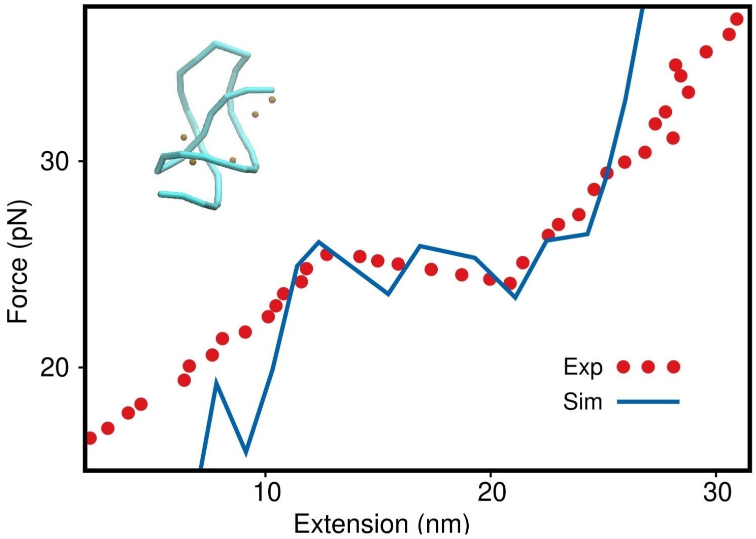

Figure S6: Force-extension curves for the MMTV pseudoknot (PDB ID: 1RNK). Experiments were conducted in the presence of 4 mM Mg^{2+} (red circles).[13] The initial 3D structure (cyan lines) contains 5 Mg^{2+} ions (yellow circles). Consistent with the experimental data, the BD simulation (blue line) was performed using the following parameters: $k_s = 0.50$ pN/nm (spring constant), $v = 190$ nm/s (speed), and $r_f = 95.9$ pN/s (loading rate).

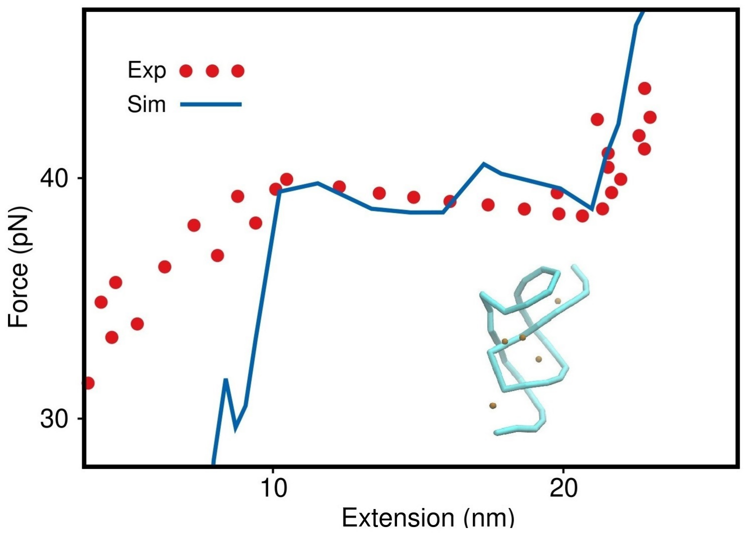

Figure S7: Force-extension curves for the PT2G32 pseudoknot (PDB ID: 2TPK). *Ibid.* to the preceding figure.

S3 Model's parameters

The model's degrees of freedom are defined based on the distances between monomers connected by phosphodiester bonds, $\mathbf{a}_i = \mathbf{r}_i - \mathbf{r}_{i-1}$, as follows:

The bending angle θ between nucleotides $i-1$, i, and $i+1$ is:

$$\theta = \arccos\left(-\mathbf{a}_{\mathrm{i+1}} \cdot \mathbf{a}_{\mathrm{i}} / |\mathbf{a}_{\mathrm{i+1}}||\mathbf{a}_{\mathrm{i}}|\right).$$

The torsion angle ϕ is given by:

$$\phi = \pi + \arctan 2\left[((\mathbf{a}_{i+1} \times \mathbf{a}_{i+2}) \times (\mathbf{a}_{i+2} \times \mathbf{a}_{i+3})) \cdot \frac{\mathbf{a}_{i+2}}{|\mathbf{a}_{i+2}|}, (\mathbf{a}_{i+1} \times \mathbf{a}_{i+2}) \cdot (\mathbf{a}_{i+2} \times \mathbf{a}_{i+3})\right].$$

The total potential energy has the explicit form:

$$\begin{aligned} U &= \sum_{bonds} \frac{1}{2} K_0 (a-a_0)^2 - A_b \exp\left[-\frac{1}{2}\left(\frac{a-a_1}{\sigma_b}\right)^2\right] \\ &+ \sum_{angles} \left(\frac{A_\theta}{\theta}\right)^6 - B_\theta \exp\left[-\frac{1}{2}\left(\frac{\theta-\theta_0}{\sigma_\theta}\right)^2\right] \\ &+ \sum_{dihedrals} -A_\phi \exp\left[-\frac{1}{2}\left(\frac{\phi-\phi_0}{\sigma_\phi}\right)^2\right] \\ &+ \sum_{i,j} \left(\frac{A_{\mu\nu}}{r}\right)^8 - B_{\mu\nu}\left[\frac{\sigma_{\mu\nu}^2}{(r-d_{\mu\nu})^2+\sigma_{\mu\nu}^2}\right] \exp\left[-\frac{1}{2}\left(\frac{\alpha-e_{\mu\nu}}{s_{\mu\nu}}\right)^2\right]. \end{aligned}$$

The potential energy between a magnesium ion and a nucleotide is given by a modulated Morse potential:[28]

$$U(d,\gamma) = \sum_{n=0}^{2} h_n \cos^n(\gamma) \left[\exp\left(-2\lambda(d-d_0)\right) - 2\exp\left(-\lambda(d-d_0)\right)\right],$$

where γ is a coupling angle, d is the distance between ion and nucleotide, h_n, d_0, and λ are adjustable parameters. The effective interaction between two magnesium ions is:[33]

$$U(d) = \frac{4e^2}{\epsilon} \frac{\exp(-\kappa_D d)}{d},$$

with d being the separation between two ions, e, ϵ, and κ_D are the elementary charge, the background dielectric constant, and the Debye inverse screening length, respectively.

The fitting parameter values for the potentials are presented in the following tables.

Table S1: Fitting parameters for the bond potential.

Parameter	Units	Value
K_0	$k_B T/Å^2$	0.73
a_0	Å	6.63
A_b	Å	4.28
a_1	$k_B T$	4.69
σ_b	Å	0.90

Table S2: Fitting parameters for the bending potential.

Parameter	Units	Value
A_θ	$(k_\mathrm{B}T\mathrm{rad})^{1/6}$	0.57
B_θ	$k_\mathrm{B}T$	2.85
θ_0	rad	2.70
σ_θ	rad	0.33

Table S3: Fitting parameters for the torsion potential.

Parameter	Units	Value
A_ϕ	$k_\mathrm{B}T$	1.15
ϕ_0	rad	3.60
σ_ϕ	rad	0.81

Table S4: Fitting parameters for the radial-angular potential.

Nt	$A_{\mu\nu}$ $((k_\mathrm{B}T\text{Å})^{1/8})$	$B_{\mu\nu}$ $(k_\mathrm{B}T)$	$e_{\mu\nu}$ (rad)	$s_{\mu\nu}$ (rad)	$d_{\mu\nu}$ (Å)	$\sigma_{\mu\nu}$ (Å)
AA	5.12	0.93	2.44	0.30	9.7	0.56
AC	5.72	0.89	2.48	0.45	10.1	0.38
AG	5.28	1.45	2.59	0.39	9.6	0.49
AU	5.92	2.65	1.79	0.26	11.6	0.43
CC	6.37	1.51	2.64	0.38	10.4	0.54
CG	5.80	2.79	1.83	0.29	11.4	0.36
CU	6.02	1.51	2.68	0.25	10.5	0.68
GG	5.90	1.68	2.75	0.40	9.2	0.72
GU	5.94	1.50	1.88	0.19	11.2	0.25
UU	5.87	1.27	2.55	0.32	10.6	0.48

Table S5: Fitting parameters for the radial-angular potential Nt-Mg.

Nt	h_0 $(k_\mathrm{B}T)$	h_1 $(k_\mathrm{B}T)$	h_2 $(k_\mathrm{B}T)$	λ (Å^{-1})	d_0 (Å)
A	0.678	0.639	2.265	0.65	5.1
C	0.986	1.102	1.462	0.70	6.2
G	1.089	0.901	1.627	0.65	6.6
U	1.436	1.722	1.503	0.60	6.5
